# External Immune Inhibitory Efficiency of External Secretions and Their Metabolic Profiling in Red Palm Weevil, *Rhynchophorus ferrugineus* (Coleoptera: Curculionidae)

**DOI:** 10.3389/fphys.2019.01624

**Published:** 2020-01-29

**Authors:** Yu-Chen Pu, Hai-Jun Xiang, Xin-Yu Liang, Yu Wang, You-Ming Hou, Lang Fu, Rui Wang

**Affiliations:** ^1^State Key Laboratory of Ecological Pest Control for Fujian and Taiwan Crops, Fujian Agriculture and Forestry University, Fuzhou, China; ^2^Fujian Provincial Key Laboratory of Insect Ecology, College of Plant Protection, Fujian Agriculture and Forestry University, Fuzhou, China

**Keywords:** antimicrobial activity, external immune defense, external secretion, metabolome, *p*-benzoquinone, red palm weevil

## Abstract

External secretions play a vital role in external immune defense. However, the functions and components of these exudates are largely unknown in the red palm weevil, *Rhynchophorus ferrugineus* (Olivier) (Coleoptera: Curculionidae). In order to determine their role in external immunity, the immunosuppressive efficacy of the secretions *in vitro* against microbes, including bacteria and fungi, was clarified. In the present study, we found that these secretions had antimicrobial activity *in vitro*, implying external immunizing potency against pathogens. Surprisingly, all liquid phases of secretions could not significantly inhibit the growth of microbes *in vitro* compared to solid phases. To explain this phenomenon, the composition and emission differentia of secretions from the exocrine glands associated with different developmental stages, secretory regions, and phases were identified and analyzed based on metabonomics techniques. A total of more than 200 compounds, including quinines, phenols, aldehydes, acids, alcohols, saccharides, ketones, esters, amines, salts, ureas, and heterocycles, were identified in the secretions of larvae and adults. The liquid phase shared a number of metabolites with the solid phase, but the emission types and amounts were significantly different in the two phases, resulting in differences in external immunological activity. Tyrosine and *p*-benzoquinone were the dominant metabolites in all of the secretions, accounting for approximately 11.29% of emissions, with the portion in the solid phase being generally higher than that in the liquid phase. Moreover, only *p*-benzoquinone was entirely significantly upregulated in the solid phase compared to the liquid phase. Therefore, metabolome analysis suggested that *p*-benzoquinone, which may potentially be developed to be a valuable marker for determining external immunity, was considered to be the main substance responsible for external immune functions. This hypothesis was further demonstrated by the antimicrobial activity of *p*-benzoquinone.

## Introduction

The invasive red palm weevil (RPW), *Rhynchophorus ferrugineus* (Olivier) (Coleoptera: Curculionidae), is considered to be one of the most devastating pests in a number of palm tree species. Many of these are widely cultivated cash crops species as well as garden ornamental trees in tropical and subtropical regions throughout the world (Cox, 1993; Hou et al., 2011; Tagliavia et al., 2014; Wan et al., 2015). In recent years, RPW infestations have tended to increase with each passing year, seriously affecting the economic and ornamental value of the plants (Al-Dosary et al., 2016). Recently, losses in global production of dates were estimated at 30% due to diseases and pests, which resulted in the Food and Agriculture Organization (FAO) designating the RPW as a category 1 pest on date palms in the Middle East (FAOSTAT, 2013). The annual loss in the Gulf region due to eradication of severely infested palms has been estimated to range from $1.74 million to $8.69 million at 1% and 5% infestations, respectively (El-Sabea et al., 2009; Al-Dosary et al., 2016). The weevil, which was initially detected in India as a pest of the coconut palm in 1891 (Lefroy, 1906; Vidyasagar, 1998) and of the date palm in 1917 (Brand, 1917), is now distributed in many countries and regions of Europe, Asia, Africa, and America (Al-Ajlan, 2008; Fiaboe et al., 2012; Giblin-Davis et al., 2013). As a result of increasing international trade during the 1980s, it was discovered in Zhongshan City, Guangdong Province, China, during the 1990s (Liu et al., 2009). Fujian and Taiwan provinces were identified as the sources of the invasion and were the hardest hit areas. From there, it rapidly spread to 13 other provinces and autonomous regions in China through infested planting material that was being transported mainly for ornamental purposes (Ju et al., 2006; Hou et al., 2011; Wang et al., 2015).

The larva of the RPW is a specialized borer that attacks the stem and the bases of the fronds, from which it can spread into the trunks, chewing up plant fibers and severely damaging the vascular system. As a result, the trees die soon after being attacked (Faleiro, 2006; Giblin-Davis et al., 2013; Al-Dosary et al., 2016). The early phase of damage is difficult to detect because the larvae remain concealed, making it impossible to be removed even after it has been discovered (Al-Dosary et al., 2016). It is understandable that the species has been classified as a quarantine pest in China (Wu et al., 2007) due to its devastating effects on palm trees. According to previous integrated pest management (IPM) strategies, there are several control methods (e.g., plant quarantine, pheromone trapping, chemical control, and cutting down and burning of infected palms) that have been used against the RPW with varying degrees of success (Faleiro, 2006; Al-Dosary et al., 2016). Among these, biological control, which is a major component of IPM that will continue to gain in popular usage in controlling pests in the future, is considered to have the most sustainable control potential. To date, a variety of pathogens, including *Beauveria bassiana* (Balsamo) Vuillemin (Verde et al., 2015), *Metarhizium anisopliae* (Metschn.) (Sun et al., 2016), *Serratia marcescens* Bizio (Pu and Hou, 2016), *Bacillus thuringiensis* Berliner (Pu et al., 2017b), and *Steinernema carpocapsae* (Weiser) (Llácer et al., 2009), have been isolated from naturally diseased dead RPW. Unfortunately, biocontrol procedures, particularly preparation of bacteria which are friendly to the environment and harmless to humans, have achieved less than ideal control efficiency when used against the RPW both in the field and in the laboratory (Pu et al., 2017a). This has led to a serious decrease in the use of biocontrol measures in attempts to control RPWs.

Various biotic and abiotic factors, including temperature, humidity, sunlight, concentration of pathogens, and the host itself, can limit and pose a challenge to the use of pathogenic organisms (Jaronski, 2010). Host immunity caused by exogenous invasion is crucial (Manachini et al., 2011; Hussain et al., 2015; Mastore et al., 2016). It is generally believed that internal immunity consisting of cellular and humoral defense mechanisms is an immediate and primary immune response of insects (Chu et al., 2013; Pu et al., 2017a). Nevertheless, another defensive strategy, external immunity, which is not viewed as an immune system component, is deployed to prevent pathogens from gaining entrance into the body (Otti et al., 2014; Pu et al., 2017a). External immune defense involves by definition any heritable trait acting outside an organism improving protection from pathogens, such as bacteria, fungi, viruses, and nematodes, or manipulating the composition of the microbial community in favor of the organism (Otti et al., 2014). This immune defensive system, which includes the cuticle and external secretions, constitutes an initial physical and chemical barrier to pathogens (Gołebiowski et al., 2008; Silva et al., 2016; Pu et al., 2017a).

These chemical defensive substances, which are a major element of external immunity in insects, are synthesized mainly through specialized exocrine glands and then released into external environments to exert immune functions *in vitro* (Gross et al., 2008; Li et al., 2013; Otti et al., 2014). Certain insect species, especially social or gregarious insects, are capable of secreting external exudates or volatiles (Otti et al., 2014; Wei et al., 2017). These external secretions consist of a number of chemical components, such as quinones (Joop et al., 2014), phenols (Qiang et al., 2006b), aldehydes (Gross et al., 2008; Ulrich et al., 2016), and lysozyme (Cotter et al., 2013). Previous research has shown that the external secretions of insects can inhibit the growth of a variety of pathogens *in vitro*, including gram-positive bacteria, gram-negative bacteria, and fungi (Li et al., 2009; Cotter et al., 2013; Pedrini et al., 2015; Ulrich et al., 2015). We speculate that these chemical mixtures may be able to initiate the insect’s immune functions to protect themselves by preventing the invasion of foreigner pathogens. Although evidence for the antimicrobial activity of such secretions exists, their role and importance in the context of immune defense is not well understood.

The accurate and timely surveillance of RPW infestation is an essential first step in developing a management strategy to control the weevils. The concealed nature of the pest usually results in failure to treat infestations in a timely manner, thereby exacerbating control efforts and allowing the pest to become established within its host (Hou et al., 2011; Wan et al., 2015). For this reason, the early detection of populations is critical. Because RPWs are known to aggregate, presumably mediated by weevil-produced or weevil-associated chemical cues, aggregation hazard characteristic has been studied in an attempt to develop a monitor or lure, and a case in point is the aggregation pheromone trap (Gunawardena and Bandarage, 1995; Muralidharan et al., 1999; Zada et al., 2002). There is sufficient evidence now to show that one of the functions of the external secretions is to cause specific behavioral responses to the insects themselves in addition to the defensive function (Pu et al., 2017a). These behavioral responses may include either an attractant (Liang, 1995; Olson et al., 2009; Weeks et al., 2013; Ulrich et al., 2015) or repellent effect (Liang, 1995; Ulrich et al., 2015).

Based on a pest’s predictability of behavioral actions when responding to its secretions, external immune secretions could potentially be used for field population density monitoring and mass trapping or be incorporated with insecticides to attract and kill the targeted insects (Ulrich et al., 2015; Pu et al., 2017a). On the other hand, many chemical secretions have repellent or irritant properties (Eisner, 1966; Blum, 1981). These may be possible candidates for repellents used to protect crops from pests (Liang, 1995; Pu et al., 2017a).

The RPW, as a gregarious pest, is confronted with the challenge of enormous microbial pressures from its environment. Under such living conditions, external immunity would seem to be more significant than internal immunity (Otti et al., 2014). To understand the role that external secretions play in external immunity through functional and metabolic analyses, we examined the inhibitory efficacy of RPW external secretions *in vitro* and then further analyzed the metabolome. Using this information, we further chose the key potential active chemical to determine and verify its antimicrobial activity. The present research discusses variation in the adaptive mechanism of invasive pests from the perspective of RPW defensive secretions and their role in external immunity. This study will contribute to our understanding of the immunity interactions that occur between the RPW and its pathogens, as well as elucidating the passive strategies employed by the RPW to avoid pathogenic infection. These results will aid in ongoing efforts to control the weevil in the field. It is also hoped that they will also provide the basis for a new concept and approach in the development of a pest inhibitor or behavioral interference agent which utilizes the immune system of the pest as a target.

## Materials and Methods

### Insects

A laboratory colony of RPW was established by collecting mature larvae, cocoons, and adults from infested date palm trees, *Phoenix canariensis* Chabaud, during July 2015, in the Xiamen City Botanical Garden (24.45°N, 118.10°E), Fujian Province, China. In the laboratory, larvae were provided with sugarcane for feeding and reared individually in Petri dishes (90 mm Ø) or 330-ml plastic bottles (after the seventh instar) at 27 ± 1°C, 75 ± 5% relative humidity (RH) and a photoperiod of constant darkness. After adults emerged, the sexes were paired, with each pair placed into a 330-ml plastic bottle with perforated lid, and fed with fresh sugarcane under the same conditions as larvae except for a 12:12-h (L:D) photoperiod. Subsequently, eggs were removed and transferred to moist absorbent cotton in Petri dishes (90 mm Ø).

### Immune Challenge for Larvae and Adults

Several viable bacterial pathogens were previously isolated and identified from RPWs (Pu and Hou, 2016). Of these, *B. thuringiensis* HA (Pu et al., 2017b) was used as the pathogen to challenge larvae and adults in this study. Before pathogenic challenges were conducted, we sterilized the surface of tested insects with alcohol-dampened cotton. To avoid the invasion of pathogens into the body cavity as much as possible to thereby provoke external immunity and weaken internal immune response, we infected larvae between the intersegmental membranes and adults between the head and thorax. Infection was achieved through the septic injury method with a 0.1-mm Minutien pin dipped into a 2-ml suspension of *B. thuringiensis*. The bacteria were cultured at 30°C and shaken overnight at 200 rpm for 12 h with OD_600_ = 1 on a nutrient broth (NB) medium (peptone 10 g, beef extract powder 3 g, NaCl 5 g, distilled water 1,000 ml, pH 7.2). Piercing was carried out in such a way as to minimize hemolymph loss with just the tip of the needle penetrating the cuticle, and in the majority of cases, there was no bleeding. After the challenge of the external immune system, we contacted the infected area with an inoculation needle and then applied it on a glass slide containing a drop of distilled water. The glass slide was placed under an optical microscope to check whether insects were successfully infected or not through observing the presence or absence of *B. thuringiensis*.

### Collection of External Secretion Samples

Fourth-instar larvae (*n* = 90) and young virgin adults (7-day-old post-adult eclosion, *n* = 126) were randomly and averagely assigned to one of six experimental replications, respectively. The immunity treatment of bacteria challenge was designed as above to challenge the personal external immune defensive system. Collection of external secretions was according to the method described by Turlings et al. (1993). In order to avoid contamination of samples by microorganisms, this process was accomplished on a precleaned beach. Following immune treatment, larvae and adults of RPW were clamped lightly with forceps, forcing them to secrete a trace of exudates on sterile disposable polyethylene (PE) gloves. These secretions were then drawn up with a 10-μl pipette gun. Three types of secretory structures, including the larval oral cavity, larval abdominal anus, and abdominal anus of adults, produced observable exudates. We have referred to these secretions as oral secretions of larvae, abdominal secretions of larvae, and abdominal secretions of adults, respectively. With the above method, secretions from these three sources were collected separately (100 μl or 100 mg) and stored in sterilized 1.5-ml Eppendorf tubes. Secretions were then centrifuged at 10,000 rpm for 10 min at 4°C after 100 μl of sterile distilled water added to the tube. The hydrosoluble supernatant was transferred separately into a new sterilized tube, and the remaining insoluble precipitate rinsed three times. Stoste samples (crude extracts), liquid-phase samples (supernatants), and solid-phase samples (precipitates) were obtained using this process. All secretion samples were stored at −80°C to be further used in subsequent experiments.

### Microbial Species Used in Inhibition Experiments

Two species of indicative bacteria, a gram-positive bacterium (*Staphylococcus aureus*) (Nanjing Biotechnology Co., Ltd., Nanjing, China) and a gram-negative bacterium (*Escherichia coli* DH5α) (Beijing TransGen Biotech Co., Ltd., Beijing, China), were used for evaluating the antimicrobial activity of the secretions *in vitro*. Two additional pathogenic microorganisms, including a bacterium (*B. thuringiensis*) and a fungus (*M. anisopliae*), were also used in the inhibition experiments.

*S. aureus* and *E. coli* were both inoculated by being added to a 50-ml Erlenmeyer flask containing 20 ml of Luria-Bertani (LB) liquid medium (tryptone 10 g, NaCl 10 g, yeast extract 5 g, distilled water 1,000 ml, pH 7.2) and then shaken at 200 rpm at 37°C until OD_600_ = 0.6. *B. thuringiensis* was cultured under the same methods as *S. aureus* and *E. coli*, except that NB medium was used at 30°C. Following 1,000 × dilution, the final concentration of 10^4^ colony-forming unit (CFU)/ml was obtained.

*M. anisopliae* was grown on potato dextrose agar (PDA) plates (potato extract powder 5 g, glucose 20 g, agar 15 g, distilled water 1,000 ml, pH 6.0) and incubated at 25°C for 7 days. Conidia were harvested by scraping colonies with a disposable sterile blade. The conidial clumps were then suspended in sterile distilled water containing 0.01% Tween 80. The concentration of spore suspension was determined using a hemocytometer and adjusted to 10^4^ conidia/ml.

### Verification of External Immune Function of Secretions

To measure the intensity of the external immune defense and verify the external immune function of the secretions, we followed the protocol described by Li et al. (2009), by measuring the diameter of the inhibition zones produced by RPW external secretions in a lawn of bacterial growth on LB agar medium (tryptone 10 g, NaCl 10 g, yeast extract 5 g, agar 15 g, distilled water 1,000 ml, pH 7.2) plates or fungal growth on PDA medium plates. Stoste samples and solid-phase samples were dissolved in 100 μl of methanol (Shanghai Sinopharm Chemical Reagent Co., Ltd., Shanghai, China) beforehand, while liquid-phase samples were diluted in 100 μl of sterile distilled water. Sterile 6-mm circular filter papers were impregnated with solutions of either secretion samples or control groups until saturated. Controls consisted of filter papers exposed to sterile water (negative control), methanol (negative control), or 50 μg/ml of tetracycline (positive control). After drying, the filter papers were placed in medium plates containing 50 μl of bacterial suspensions (10^4^ CFU/ml) or fungal suspensions (10^4^ conidia/mL) that had previously been uniformly spread over the surface using a sterile spreader. Plates for growing bacteria were kept at 37°C (*S. aureus* and *E. coli*) or 30°C (*B. thuringiensis*) and incubated for 12 h. Plates for growing fungi were maintained at 25°C for 72 h. Antimicrobial activity was indicated by a clear zone in the plate. Each sample was replicated technically three times.

To further determine the minimum inhibitory concentration (MIC) of secretion stostes required for *S. aureus*, *E. coli*, *B. thuringiensis*, and *M. anisopliae* inhibition, the microbial suspension (10^4^ CFU/ml or 10^4^ conidia/ml) and diluents of stoste (methanol as a control) diluted with methanol into 500.00, 250.00, 125.00, 62.50, and 31.25 μl/ml using the twofold dilution method were mixed with equal volumes and cultured for 2 h for bacteria or 24 h for fungi at the temperatures described above. After 2 or 24 h, an intermixture of 50 μl was uniformly spread over the entire surface of the agar medium. All plates were kept at the same conditions as above for 12 or 72 h. The minimal concentration was needed to suppress all microbial growth.

### The Metabonomics Analysis of RPW External Secretions

Liquid-phase and solid-phase samples of secretions were submitted to Shanghai Bioclouds Biological Technology Co., Ltd., Shanghai, China, for untargeted metabolomics detection. Six groups (A, B, C, D, E, and F) of different samples (a total of 36 samples) were examined (A: liquid phase of larval oral secretions; B: solid phase of larval oral secretions; C: liquid phase of larval abdominal secretions; D: solid phase of larval abdominal secretions; E: liquid phase of adult abdominal secretions; and F: solid phase of adult abdominal secretions). Each sample of 30 mg was accurately weighed in a 1.5-ml centrifuge tube. The L-2-chlorophenylalanine (0.3 mg/ml) of 20 μl (internal standard), which was prepared using methanol, and the 600 μl methanol–water solution (v:v = 4: 1) were then added successively. After two small steel balls were added, the samples were placed in the refrigerator at −80°C for 2 min and then grinded at 60 Hz for 2 min. It was then distilled in an ice water bath using ultrasonic extraction and statically placed in −20°C for 30 min. A total of 400 μl of supernatant, which was volatilized and dried by using a centrifuge concentrating dryer, was transferred to a glass derivative bottle after the sample was centrifuged at 13,000 rpm for 15 min at 4°C. When the methoxamine hydrochloride pyridine (15 mg/ml) of 80 μl was added to a glass derivative bottle, followed by 2 min of vortex concussion, oximation reaction was performed for 90 min at 37°C in a concussion incubator. Eighty microliters of bis(trimethylsilyl)trifluoroacetamide (BSTFA) containing 1% trimethylchlorosilane (TMCS) and 20 μl of hexane were added and allowed to react for 60 min at 70°C after 2 min of vortex concussion. All samples were finally left at room temperature for 30 min to complete the metabolome analysis. The quality control (QC) sample consisted of isopycnic extract in all samples. The volume of each QC was the same as that of the sample.

We determined and quantified the chemical compound profiles of the secretions using gas chromatography–mass spectrometry (GC-MS). Secretion extracts were run on a 7980A gas chromatograph coupled to a 5975C mass spectrometer (Agilent, United States). One microliter of each sample was injected onto an HP-5MS capillary column (30 m × 0.25 mm × 0.25 μm, Agilent J&W Scientific, Folsom, CA, United States) using an Agilent G6500 CTC PAL autosampler. The injection was run in the pulsed splitless mode. The inlets were set at 280°C with a constant flow rate of 6.0 ml/min of helium as carrier gas. The GC oven initial temperature profile began at 60°C, ramping at 8°C/min to 125°C, and then increased to 190°C at 10°C/min to 210°C at 4°C/min, followed by a programed rate at 20°C/min to 310°C, and 310°C was finally maintained for 8.5 min. The electron impact ion source (EI, 70 eV) was operated in full-scan mode (*m*/*z* 50–600) with the MS source temperature at 230°C and MS Quad at 150°C. ChromaTOF 4.34 (LECO, St. Joseph, MI, United States) software was used to preprocess the original GC-MS data. Metabolites were identified by comparing their retention times (RTs) with the synthetic standards on the same column. The referenced mass spectra were from the Fiehn Library linked to ChromaTOF 4.34 and the NIST 11 Library (Scientific Instrument Services, Inc., Ringoes, NJ, United States). A three-dimensional data matrix in CSV file format was obtained. This included sample information, RT, metabolite name, and mass spectrum response intensity, but the internal standard and any known pseudo-positive peaks, such as peaks caused by noise, column bleed, and BSTFA derivatization procedure, were removed. Peak merging and deredundancy were also performed. The peak area normalization method was used to normalize the response intensity of the sample mass spectrum peak to generate the normalized data matrix. Multidimensional statistics and metabolic pathways focusing on total metabolites or differential metabolites (A vs. B; C vs. D; E vs. F; A vs. C; A vs. E; C vs. E; B vs. D; B vs. F; and D vs. F) were further analyzed to identify the role that external secretions play in the external immunity of RPWs.

### Determination of the Antimicrobial Potency of *p*-Benzoquinone

White crystal powder of *p*-benzoquinone (PBQ) was obtained from J&K Scientific Ltd. (Beijing, China). The inhibitory activities of 10 mg/ml of PBQ against *S. aureus*, *E. coli*, *B. thuringiensis*, and *M. anisopliae* were validated and compared by determining the diameter of the inhibition zones. MIC was then determined by diluting 10 mg/ml of PBQ solution with sterile water into 10.00, 5.00, 2.50, 1.25, 0.64, 0.32, 0.16, 0.08, 0.04, 0.03, 0.02, and 0.01 mg/ml, respectively.

### Data Analysis

SPSS 21.0 (IBM Inc., Chicago, IL, United States) statistical analysis software was used to process all data, and the SigmaPlot 12.0 (Systat Inc., San Jose, CA, United States) drawing program was used for constructing graphs. Comparisons for the inhibition zone diameter data were performed using one-way ANOVA as well as Tukey’s honestly significant difference (HSD) test for multiple comparisons (α = 0.05).

Orthogonal partial least squares discriminant analysis (OPLS-DA) was carried out to separate profiles of external secretions from the oral cavity and the abdomen from the two different developmental stages and sample phases and to visualize alterations among experimental groups, after mean centering and unit variance scaling, using SIMCA-P 14.1 (Umetrics AB, Umeå, Sweden). Variable importance in the projection (VIP), which ranks the overall contribution of each variable to the OPLS-DA model, was calculated. Variables with both VIP > 1.0 and *P* < 0.05 are considered relevant for group discrimination and the most influential for the model (Jansson et al., 2009; Wei et al., 2014). The bioinformatics cloud platform OmicsShare tools 3.0^1^ (Gene *Denovo* Biotechnology Co., Ltd., Guangzhou, China) was used to screen all differential metabolites among samples and cluster analysis of differential metabolites, such as volcano plot and heatmap. To further interpret the biological significance associated with external immunity, we applied the Kyoto Encyclopedia of Genes and Genomes (KEGG) database to link these metabolites to metabolic pathways.

## Results

### Immunosuppressive Efficacy of External Secretions *in vitro*

**FIGURE 1:**
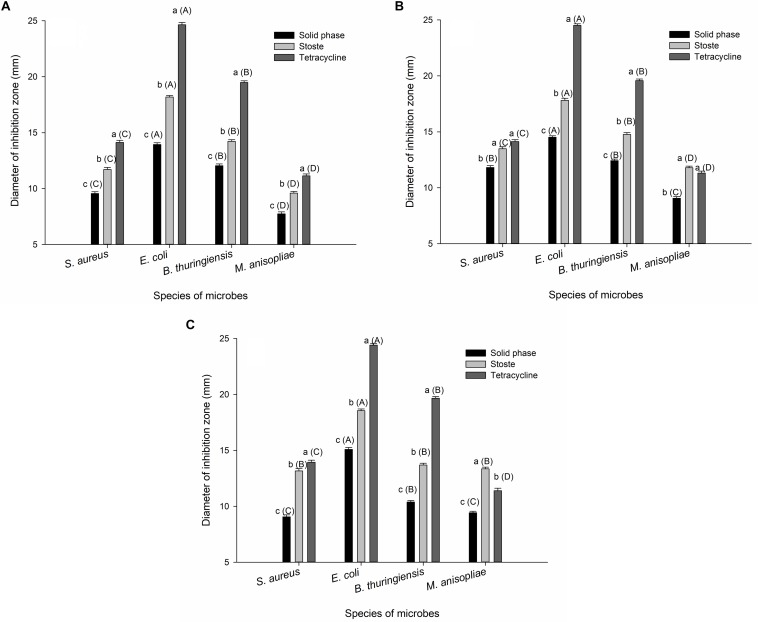
The diameter of the inhibition zone against microbes formed by **(A)** larval oral secretions, **(B)** larval abdominal secretions, and **(C)** adult abdominal secretions. The graph shows mean ± standard error. Different lowercase letters outside parentheses indicate significant difference among different treatments within the same tested microbe, while different uppercase letters inside parentheses indicate significant difference among different species of microbes under the same treatment, according to Tukey’s honestly significant difference (HSD) test of one-way ANOVA (*P* < 0.05).

Control filter papers treated with sterile water or methanol showed no inhibition of microbial growth, while the external secretions, as well as control filter papers treated with tetracycline, successfully inhibited growth of bacteria and fungi *in vitro*, including *S. aureus*, *E. coli*, *B. thuringiensis*, and *M. anisopliae*. Similar results were obtained in each of the different types of secretions tested. Both the stoste and solid-phase samples formed obvious inhibition zones, while the liquid-phase samples were much less evident. The extent of antimicrobial activity of stoste against *S. aureus* (larval oral secretions: *F*_2_,_51_ = 170.793, *P* < 0.001; larval abdominal secretions: *F*_2_,_51_ = 48.768, *P* < 0.001; adult abdominal secretions: *F*_2_,_51_ = 182.705, *P* < 0.001), *E. coli* (larval oral secretions: *F*_2_,_51_ = 1025.156, *P* < 0.001; larval abdominal secretions: *F*_2_,_51_ = 1049.238, *P* < 0.001; adult abdominal secretions: *F*_2_,_51_ = 833.978, *P* < 0.001), *B. thuringiensis* (larval oral secretions: *F*_2_,_51_ = 600.013, *P* < 0.001; larval abdominal secretions: *F*_2_,_51_ = 601.721, *P* < 0.001; adult abdominal secretions: *F*_2_,_51_ = 984.095, *P* < 0.001), or *M. anisopliae* (larval oral secretions: *F*_2_,_51_ = 99.362, *P* < 0.001; larval abdominal secretions: *F*_2_,_51_ = 86.678, *P* < 0.001; adult abdominal secretions: *F*_2_,_51_ = 127.443, *P* < 0.001) was all significantly higher than that in the solid phase (Figure 1). The results also demonstrated that the inhibition zones in *E. coli* formed by the solid phase (larval oral secretions: *F*_3_,_68_ = 281.970, *P* < 0.001; larval abdominal secretions: *F*_3_,_68_ = 237.387, *P* < 0.001; adult abdominal secretions: *F*_3_,_68_ = 318.144, *P* < 0.001) or stoste (larval oral secretions: *F*_3_,_68_ = 540.130, *P* < 0.001; larval abdominal secretions: *F*_3_,_68_ = 224.681, *P* < 0.001; adult abdominal secretions: *F*_3_,_68_ = 228.501, *P* < 0.001) were both significantly larger than those in *S. aureus*, *B. thuringiensis*, and *M. anisopliae* (Figure 1).

Using the MIC of secretion stoste that exhibited inhibition of *S. aureus*, *E. coli*, *B. thuringiensis*, and *M. anisopliae in vitro*, we found that the degree of inhibition increased in all of the tested microbes when concentrations of the secretions were increased. The antimicrobial activity of larval oral secretions and adult abdominal secretions had similar inhibitory effects (MIC of 125.00, 62.50, 62.50, and 250.00 μl/ml, respectively), with both being greater than the larval abdominal secretions (MIC of 250.00, 125.00, 125.00, and 500.00 μl/ml, respectively) (Table 1).

**TABLE 1:** Suppression of microbes exposed to secretions at various concentrations.

**Concentration (μl/ml)**	**Larval oral secretions**				**Larval abdominal secretions**				**Adult abdominal secretions**			
	***Sa***	***Ec***	***Bt***	***Ma***	***Sa***	***Ec***	***Bt***	***Ma***	***Sa***	***Ec***	***Bt***	***Ma***
500.00	−	−	−	−	−	−	−	−	−	−	−	−
250.00	−	−	−	−	−	−	−	+	−	−	−	−
125.00	−	−	−	+	+	−	−	+	−	−	−	+
62.50	+	−	−	+	+	+	+	+	+	−	−	+
31.25	+	+	+	+	+	+	+	+	+	+	+	+
0.00	+	+	+	+	+	+	+	+	+	+	+	+

*“+”: growth; “−”: inhibition; “*Sa*”: *Staphylococcus aureus*; “*Ec*”: *Escherichia coli*; “*Bt*”: *Bacillus thuringiensis*; “*Ma*”: *Metarhizium anisopliae*.*

### Chemical Components of External Immune Secretions From RPW

A total of more than 200 compounds, based on total ion current (TIC) chromatograms (Supplementary Figure S1), were identified in the secretions of RPW larvae and adults. Specifically, the components from six different types of samples (A to F) contained 259, 183, 206, 237, 235, and 132 chemical compounds, respectively. These compounds were mainly enriched quinones (PBQ, hydroquinone, 1-hydroxyanthraquinone, and acenaphthenequinone), phenols (*m*-cresol, phloroglucinol, 3-phenylcatechol, 4-vinylphenol, 4-nitrocatechol, α-tocopherol, 1,2,4-benzenetriol, 2-aminophenol, and 5-methylresorcinol) and aldehydes (octanal, phenylacetaldehyde, butyraldehyde, glutaraldehyde, farnesal, 2,5-dihydroxybenzaldehyde, *trans*-3,5-dimethoxy-4-hydroxycinnamaldehyde, and succinate semialdehyde). In addition to the three categories mentioned above, the following components were also present: acids, alcohols, saccharides, ketones, esters, amines, salts, ureas, and heterocycles (Table 2). Among the 51 predominant secretory chemicals found in either single or multiple types of samples, PBQ and tyrosine were the most common in all of the secretions, accounting for 11.29% of major secretion emissions, with the proportion in the solid phase being generally higher than that in the liquid phase (Table 2). Liquid-phase substances of larval oral secretions, larval abdominal secretions, and adult abdominal secretions were quantitatively characterized by having large amounts of β-mannosylglycerate (32.01%), lactic acid (22.09%), and urea (28.04%). Large amounts of tyrosine (15.73%), β-mannosylglycerate (18.35%), and uric acid (13.88%) were found in the solid phase (Table 2).

**TABLE 2:** Major chemical components of red palm weevil-associated mixtures, collected from the external secretions of larvae and adults.

**Chemical compounds^a^**	**Molecular formula**	**Mass**	**Retention time (min)**	**Average mass spectrum response intensity**					
				**A**	**B**	**C**	**D**	**E**	**F**
β-Mannosylglycerate	C_9_H_16_O_9_	204	14.86	2,418.38**	472.42*	1,693.12*	1,591.73**	301.22*	2.69
Putrescine	C_4_H_12_N_2_	174	15.76	760.38*	482.02*	59.57	197.82*	332.54*	27.56
1-Methylhydantoin	C_4_H_6_N_2_O_2_	258	7.03	452.86*	1,181.75*	981.32*	366.40*	12.22	134.14*
Glucoheptonic acid	C_7_H_14_O_8_	217	16.59	372.23*	10.98	109.31*	11.60	ND	ND
Glucose-1-phosphate	C_6_H_13_O_10_P	217	8.88	366.79*	97.22*	61.78	54.58	79.81*	69.31
Gluconic acid	C_6_H_12_O_7_	73	19.54	324.90*	549.30*	39.65	1,287.21*	0.09	ND
Glycine	C_2_H_5_NO_2_	84	18.09	243.34*	50.31	40.72	35.39	5.33	5.90
1-Kestose	C_19_H_34_O_16_	217	16.50	225.88*	ND	1.50	0.54	ND	ND
5-Methoxytryptamine	C_11_H_14_N_2_O	174	7.65	222.29*	4.55	449.38*	13.83	12.77	5.11
Galactonic acid	C_6_H_12_O_7_	73	16.25	219.12*	10.50	26.15	12.62	5.70	ND
Lactic acid	C_3_H_6_O_3_	147	6.10	194.29*	149.95*	1,810.50**	374.75*	15.53	539.04*
Galactinol	C_12_H_22_O_11_	204	22.35	174.67*	10.14	25.42	24.78	2.44	ND
Oxoproline	C_5_H_7_NO_3_	156	13.09	169.48*	93.43*	89.49*	45.04	155.78*	12.78
itp-Benzoquinone	C_6_H_4_O_2_	121	5.24	150.48*	407.40*	115.60*	297.64*	213.26*	1,025.70*
Threonic acid	C_4_H_8_O_5_	73	13.55	121.60*	ND	279.10*	11.74	18.19	1.16
Dehydroascorbic acid	C_6_H_6_O_6_	173	20.57	111.54*	10.87	ND	91.10*	ND	ND
2-Hydroxypyridine	C_5_H_5_NO	152	5.81	107.03*	234.60*	173.27*	76.89	135.69*	10.30
Tyrosine	C_9_H_11_NO_3_	226	12.40	106.73*	1,242.64**	103.95*	515.45*	923.18*	390.43*
Citric acid	C_6_H_8_O_7_	273	16.81	96.91*	ND	2.05	8.30	4.62	ND
Glycerol	C_3_H_8_O_3_	205	9.57	93.12*	279.64*	278.22*	334.93*	265.18*	977.24*
Phosphate	H_3_PO_4_	299	9.53	49.91	418.05*	217.15*	364.71*	485.50*	399.34*
Hydroxylamine	H_3_NO	249	7.00	13.11	156.52*	131.77*	42.21	81.91*	351.44*
4-Aminobutyric acid	C_4_H_9_NO_2_	174	13.20	24.06	165.06*	7.46	201.90*	33.46	ND
Alanine	C_3_H_7_NO_2_	116	7.95	0.40	12.05	73.32	503.80*	32.77	83.79
Dehydroabietic acid	C_20_H_28_O_2_	239	10.70	50.56	144.02*	ND	ND	10.06	28.40
Glutamine	C_5_H_10_N_2_O_3_	155	5.28	50.87	30.67	83.99	42.28	58.08	369.13*
Aminooxyacetic acid	C_2_H_5_NO_3_	160	7.40	84.43	220.94*	129.79*	449.21*	75.75	687.10*
Ethanolamine	C_2_H_7_NO	174	9.47	35.11	31.53	19.16	78.76	177.40*	ND
scd-Arabitol	C_5_H_12_O_5_	217	15.48	70.33	66.74	301.61*	657.61*	0.44	ND
Dithioerythritol	C_4_H_10_O_2_S_2_	221	5.02	18.27	350.72*	214.81*	14.58	237.38*	230.75*
Threitol	C_4_H_12_O_4_	217	12.81	38.80	5.62	173.04*	38.48	35.05	0/68
Hydroquinone	C_6_H_6_O_2_	239	10.70	ND	ND	143.54*	67.72	ND	ND
Valine	C_5_H_11_NO_2_	154	15.42	59.58	109.49*	89.52*	167.47*	27.52	197.79*
*N*-Acetyl-*N*-formyl-5- Methoxykynurenamine	C_13_H_16_N_2_O_4_	234	6.12	ND	100.77*	87.70*	2.10	4.13	125.54*
2-Aminoethanethiol	C_2_H_7_NS	174	6.06	0.87	553.80*	ND	166.62*	319.17*	254.77*
Leucine	C_6_H_13_NO_2_	158	9.55	ND	57.79	29.89	148.21*	11.58	33.11
Mannitol	C_6_H_14_O_6_	205	18.43	6.04	13.83	25.69	96.99*	3.99	2.97
Aminomalonic acid	C_3_H_5_NO_4_	84	7.22	5.43	18.80	8.94	84.57*	27.33	ND
Isoleucine	C_6_H_13_NO_2_	158	9.88	9.65	23.39	11.77	82.34*	11.87	14.27
Urea	CH_4_N_2_O	189	9.11	ND	ND	18.22	8.61	2,355.66**	9.57
Noradrenaline	C_12_H_17_NO_9_	174	16.51	3.83	ND	ND	3.44	930.38*	171.01*
4-HydroxymaNDelic acid	C_8_H_8_O_4_	267	16.09	ND	ND	ND	ND	226.25*	ND
Shikimic acid	C_7_H_10_O_5_	204	6.37	0.50	1.30	5.92	8.03	225.13*	ND
*trans*-4-Hydroxy-L-proline	C_5_H_9_NO_3_	140	13.14	ND	ND	6.96	0.31	202.65*	ND
Uric acid	C_5_H_4_N_4_O_3_	172	20.90	ND	ND	11.15	2.64	197.87*	1,100.24**
Hypoxanthine	C_5_H_4_N_4_O	265	10.65	0.93	5.24	0.69	23.87	79.56*	0.90
Creatine	C_4_H_9_N_3_O_2_	147	6.53	27.30	24.20	10.85	46.33	9.07	175.26*
Palmitic acid	C_16_H_32_O_2_	313	20.31	51.03	47.16	8.55	2.45	22.09	151.10*
Benzoic acid	C_7_H_6_O_2_	179	9.21	21.41	54.94	42.76	16.38	34.26	135.36*
*N*-Ethylglycine	C_4_H_9_NO_2_	202	8.67	0.10	1.19	0.92	ND	ND	102.16*
Guanine	C_5_H_5_N_5_O	171	21.15	ND	0.14	0.44	1.28	1.47	99.23*

^*a*^Compounds were identified by comparison of their retention time and mass spectrometry spectra with those of reference compounds in the Fiehn and NIST 11 libraries. A: liquid phase of larval oral secretion samples; B: solid phase of larval oral secretion samples; C: liquid phase of larval abdominal secretion samples; D: solid phase of larval abdominal secretion samples; E: liquid phase of adult abdominal secretion samples; F: solid phase of adult abdominal secretion samples. ND, not detectable (absent or peak too small to determine composition). ** The compound with the highest content in each sample. * Those compounds with content ranking in the top 20 in each sample.

Qualitative and quantitative analyses of metabolites additionally suggested that there was a substantial quantity of amino acids encoding proteins, including glycine, threonic acid, tyrosine, alanine, glutamine, valine, leucine, and isoleucine, in the external secretions produced by RPWs (Table 2). We also identified a small amount of phenylalanine, tryptophan, aspartic acid, asparagine, glutamic acid, lysine, serine, cysteine, proline, and histidine.

**FIGURE 2:**
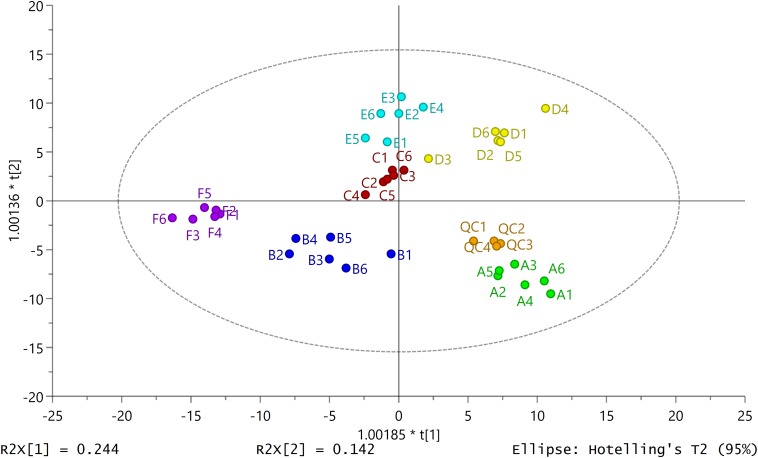
Secretion patterns produced by secretions from different body structures in red palm weevil larvae and adults. Two-dimensional orthogonal projection to latent structures discriminant analysis (2D OPLS-DA) score plot separating different types of secretion samples (**A:** liquid phase of larval oral secretions; **B:** solid phase of larval oral secretions; **C:** liquid phase of larval abdominal secretions; **D:** solid phase of larval abdominal secretions; **E:** liquid phase of adult abdominal secretions; **F:** solid phase of adult abdominal secretions; **QC**: quality control samples). Each dot with the same color represents the secretory profile of six biological replicates (*N* = 36) except QC samples (*N* = 4). The ellipse defines Hotelling’s T2 confidence region (95%).

The score plot of OPLS-DA to identify the variations found in the external secretions obtained from RPWs at different developmental stages and phases and from different secretory structures was constructed from data obtained by running GC-MS of the secretory compounds. The secretory profiles differed significantly and fell into six distinct clusters, illustrating the increasing divergence from different types of secretions and different phases (Figure 2). OPLS-DA showed a model with seven components [model statistics: *N* = 40, *K* = 259, R2X(cum) = 0.695, R2Y(cum) = 0.899, Q2(cum) = 0.781]. The components clearly separated the data points for the three types of secretions and two phases and contributed 78% of the total variance (Figure 2). The importance of the variables (compounds) in the clusters was determined based on the VIP. Compounds with a VIP > 1.0 contributed heavily to the separation of groups. Glucoheptonic acid with a VIP of 1.33 would appear to be the most important compound among the different types of secretions and phase-related chemicals (Supplementary Figure S2).

### Screening and Analysis of Differential Metabolites

It was evident from the above results that all solid phases of external secretions could significantly inhibit the growth of microbes *in vitro*, which plays an important role in external immunity. Liquid phases, however, did not (Figure 1), while the antimicrobial activity of different types of secretions also varied (Table 1). We wondered whether these results could be verified by differences in the chemical components of the secretions and in the metabolic phenotypes of individuals.

**FIGURE 3:**
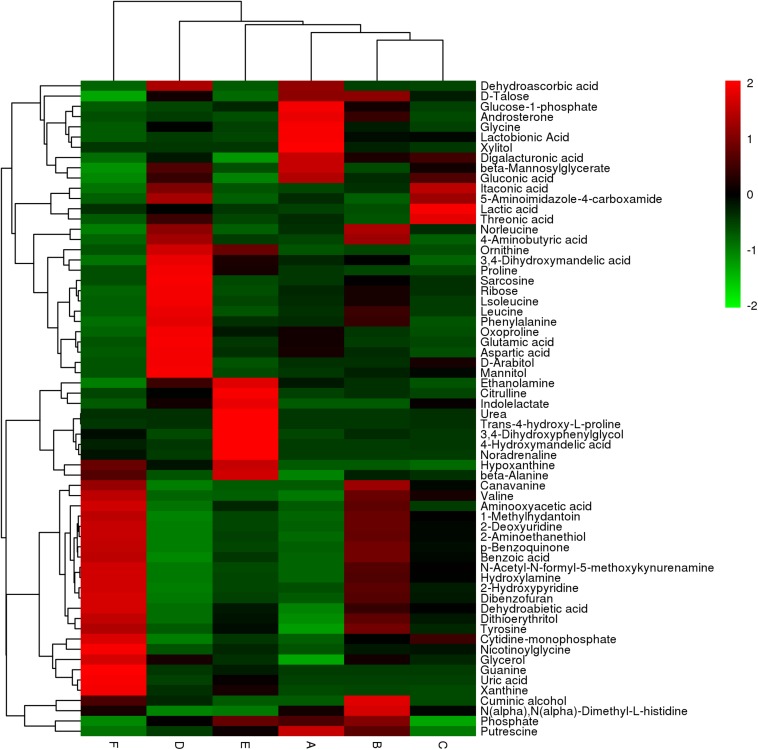
The hierarchical clustering heatmap showing changes in 64 representative differential metabolites between different types of secretions and their different phases in the six groups (**A:** liquid phase of larval oral secretions; **B:** solid phase of larval oral secretions; **C:** liquid phase of larval abdominal secretions; **D:** solid phase of larval abdominal secretions; **E:** liquid phase of adult abdominal secretions; **F:** solid phase of adult abdominal secretions). Each box represents a metabolite. The color, which shows the average normalized intensities of each metabolite, changing from green (downregulated) to red (upregulated) indicates higher levels of the metabolites. Each row indicates the levels of a specific metabolite in different samples, and each column indicates the levels for all of the differential metabolites in an individual sample. The upper dendrogram represents the cluster analysis results of different samples, and the left dendrogram represents the cluster analysis results of different metabolites. The colors correspond to the relative metabolite areas that were converted to *Z*-scores.

Differential metabolites were screened and analyzed to further clarify the immune active ingredients and key factors. We chose a total of 64 metabolites that were differentially expressed between the solid-phase groups relative to the liquid-phase groups and between any two types of secretions of the same phase. These alterations of representative metabolites are depicted as a heatmap (Figure 3), which shows two clades formed by six types of secretion samples plus two large clusters formed by differential metabolites. The metabolites in the larval oral secretions and adult abdominal secretions were the most similar in the liquid phases, while the larval abdominal secretions and adult abdominal secretions produced the most similar metabolites in the solid phases (Figure 3). In different phases, the metabolites were the closest only in the larval oral secretions (Figure 3).

**FIGURE 4:**
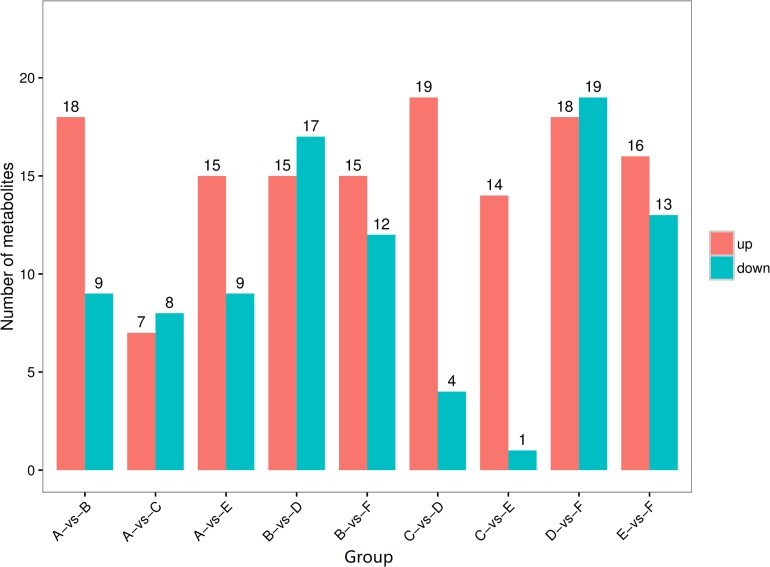
The number of differential metabolites presents in different external immunity secretion types and phases of red palms weevils. Red columns total the number of upregulated differential metabolites, and blue columns total the number of downregulated differential metabolites. **(A)** Liquid phase of larval oral secretions; **(B)** solid phase of larval oral secretions; **(C)** liquid phase of larval abdominal secretions; **(D)** solid phase of larval abdominal secretions; **(E)** liquid phase of adult abdominal secretions; **(F)** solid phase of adult abdominal secretions.

The differential metabolites that were significantly changed between any two types of secretions of the same phase or between the solid phase and the liquid phase of the same type of secretion are shown visually as volcano plots (Supplementary Figure S3). Compared with the liquid phases, the number of upregulated differential metabolites in the solid phases of the larval oral secretions, larval abdominal secretions, and adult abdominal secretions was 18, 19, and 16, respectively, whereas the number of downregulated differential metabolites was only 9, 4, and 13, respectively (Figure 4). The number of upregulated compounds exceeding the downregulated ones suggests that some substance(s) in the external secretions, especially in these upregulated differential metabolites, may play a key role in external immunity against pathogens.

Five mutual altered metabolites—oxoproline, PBQ, valine, *N*-acetyl-*N*-formyl-5-methoxykynurenamine, and hydroxylamine—were found between the liquid and solid phases in different types of secretions. However, compared to that in the liquid phase, only PBQ was entirely and significantly upregulated in the solid phase. The upregulated multiples of the metabolite in larval oral secretions, larval abdominal secretions, and adult abdominal secretions were 2.92, 2.44, and 2.96, respectively (Table 3). A total of 11 mutual altered metabolites were found in every solid phase in each pair of secretion types. The amount of PBQ varied considerably in the different secretion types. It was downregulated 0.24-fold in larval oral secretions versus larval abdominal secretions; in larval oral secretions versus adult abdominal secretions, it was upregulated 1.25-fold; and in larval abdominal secretions versus adult abdominal secretion, it was upregulated 5.14-fold (Table 3). The average mass spectrum response intensity of PBQ was gradually and sequentially enhanced in the following order: solid phase of adult abdominal secretions (1,025.70), solid phase of larval oral secretions (407.40), solid phase of larval abdominal secretions (297.64), liquid phase of adult abdominal secretions (213.26), liquid phase of larval oral secretions (150.48), and liquid phase of larval abdominal secretions (115.60) (Table 1). No other common differential metabolites were characterized between the liquid phases. Four of the metabolites (*N*-acetyl-*N*-formyl-5-methoxykynurenamine, oxoproline, PBQ, and hydroxylamine) were further identified as common differentially expressed chemicals (Table 3). These results suggested that not only is PBQ likely to be a major immune active substance in RPW, but other components may also synergistically help to regulate their external immune efficacy against pathogens.

**TABLE 3:** Comparison of the mutual altered metabolites from red palm weevil secretion samples.

**Compounds**	**FC^c^**	***P*^d^**	**VIP^e^**	**FC^c^**	***P*^d^**	**VIP^e^**	**FC^c^**	***P*^d^**	**VIP^e^**
			
**Different phases^a^**	**A versus B**			**C versus D**			**E versus F**		
*N*-Acetyl-*N*-formyl-5-methoxykynurenamine*	3.22↑^f^	3.61 × 10^–4^	1.48	0.33↓^*g*^	4.66 × 10^–2^	1.27	3.51↑	7.67 × 10^–6^	1.54
Oxoproline*	0.56↓	2.10 × 10^–2^	1.28	6.16↑	1.45 × 10^–3^	4.31	0.37↓	6.90 × 10^–3^	1.22
*p*-Benzoquinone*	2.92↑	6.40 × 10^–4^	2.86	2.44↑	4.88 × 10^–2^	2.08	2.96↑	1.03 × 10^–5^	2.73
Hydroxylamine*	3.31↑	3.09 × 10^–4^	1.86	0.34↓	4.91 × 10^–2^	1.54	3.52↑	7.45 × 10^–6^	1.93
Valine	10.43↑	1.47 × 10^–3^	1.73	0.24↓	4.04 × 10^–2^	1.41	6.58↑	6.47 × 10^–6^	1.65
**Solid phases^b^**	**B versus D**			**B versus F**			**D versus F**		
2-Hydroxypyridine	0.28↓	1.75 × 10^–4^	2.28	1.39↑	1.33 × 10^–2^	1.81	4.97↑	8.77 × 10^–7^	2.32
Dehydroabietic acid	0.40↓	3.58 × 10^–4^	1.58	1.60↑	6.97 × 10^–5^	1.98	3.99↑	1.11 × 10^–6^	1.88
*N*-Acetyl-*N*-formyl-5-methoxykynurenamine*	0.24↓	2.58 × 10^–4^	1.54	1.47↑	1.10 × 10^–2^	1.31	6.23↑	5.06 × 10^–7^	1.60
Oxoproline*	4.36↑	2.82 × 10^–3^	3.10	0.50↓	2.65 × 10^–2^	1.29	0.11↓	9.68 × 10^–4^	2.54
*p*-Benzoquinone*	0.24↓	2.66 × 10^–4^	3.09	1.25↑	1.18 × 10^–2^	1.58	5.14↑	9.96 × 10^–7^	2.89
2-Deoxyuridine	0.24↓	1.49 × 10^–4^	1.60	1.31↑	4.45 × 10^–2^	1.01	5.36↑	6.46 × 10^–7^	1.54
1-Methylhydantoin	0.27↓	2.06 × 10^–5^	5.29	1.25↑	1.52 × 10^–2^	3.05	4.73↑	1.88 × 10^–7^	4.92
2-Aminoethanethiol	0.25↓	1.50 × 10^–4^	3.60	1.33↑	3.22 × 10^–2^	2.35	5.24↑	1.17 × 10^–6^	3.49
Hydroxylamine*	0.23↓	2.42 × 10^–4^	1.93	1.48↑	8.70 × 10^–3^	1.68	6.39↑	3.67 × 10^–7^	2.01
Putrescine	0.36↓	6.43 × 10^–3^	2.98	0.10↓	6.97 × 10^–4^	4.45	0.28↓	6.68 × 10^–3^	1.38
Ethanolamine	2.38↑	2.95 × 10^–2^	1.09	0.03↓	1.19 × 10^–2^	1.11	0.01↓	3.61 × 10^–4^	1.16

*A: liquid phase of larval oral secretion samples; B: solid phase of larval oral secretion samples; C: liquid phase of larval abdominal secretion samples; D: solid phase of larval abdominal secretion samples; E: liquid phase of adult abdominal secretion samples; F: solid phase of adult abdominal secretion samples. ^*a*^Differential metabolites that are shared in common by the liquid and solid phases of the same secretion. ^*b*^Differential metabolites that are shared in common by the solid phases of each different secretion type. ^*c*^FC (fold change), calculated from the arithmetic mean values of each group (the treated group/the control group), shows how these selected differential metabolites varied in the treated groups (the second groups) compared to the control groups (the first groups). ^*d*^*P*-values were calculated using Student’s *t*-test. ^*e*^VIP (variable importance in the projection) values were obtained from OPLS-DA based on the annotated differential metabolites with a threshold of 1.0. ^*f*^Upward arrows indicated that the contents of corresponding metabolites were upregulated. ^*g*^Downward arrows indicated that the contents of corresponding metabolites were downregulated. Asterisks show common differential metabolites between different phases and solid phases. Primary biomarkers (differential metabolites) of external immunity were selected when both VIP values > 1.0 and *P*-values < 0.05.*

All differential metabolites were, for the most part, associated with one or more of the 32 pathways. External immunity biomarkers of RPW were significantly enriched into five metabolic pathways including arginine and proline metabolism (*P* < 0.0001); aminoacyl-tRNA biosynthesis (*P* = 0.0077); glutathione metabolism (*P* = 0.0108); phenylalanine, tyrosine, and tryptophan biosynthesis (*P* = 0.0162); and valine, leucine, and isoleucine biosynthesis (*P* = 0.0294) (Table 4). We noted that PBQ was produced by riboflavin metabolism and aminobenzoate degradation (Table 4 and Supplementary Figure S4). It can be biosynthesized by 4-carboxy-4-sulfoazobenzene, parathion, 4-nitrophenol-phosphate, or 1,2,4-benzenetriol through a series of enzyme reactions *in vivo* (Supplementary Figure S4). In addition, there were five pathways associated with tyrosine, the compound with the highest content, namely, glutathione metabolism; phenylalanine, tyrosine, and tryptophan biosynthesis; tyrosine metabolism; phenylalanine metabolism; and ubiquinone and other terpenoid-quinone biosynthesis (Table 4).

**TABLE 4:** KEGG pathways of differential metabolites enriched in external immune defense which was aimed at secretions of red palm weevils.

**Metabolic pathway**	**Count**	**Matched metabolites**	***P***	**−Log(*P*)**	**Impact**	**URL**
Arginine and proline metabolism	9	Citrulline; aspartic acid; ornithine; proline; glutamic acid; putrescine; urea; *trans*-4-hydroxy-L-proline; 4-aminobutyric acid	8.09 × 10^–5^	9.42	0.48	http://www.genome.jp/kegg-bin/show_pathway?dme00330
Aminoacyl-tRNA biosynthesis	9	Phenylalanine; glycine; aspartic acid; valine; isoleucine; leucine; tyrosine; proline; glutamic acid	7.70 × 10^–3^	4.87	0.00	http://www.genome.jp/kegg-bin/show_pathway?dme00970
Glutathione metabolism	5	Glycine; oxoproline; ornithine; glutamic acid; putrescine	1.08 × 10^–2^	4.53	0.13	http://www.genome.jp/kegg-bin/show_pathway?dme00480
Phenylalanine, tyrosine, and tryptophan biosynthesis	2	Phenylalanine; tyrosine	1.62 × 10^–2^	4.12	1.00	http://www.genome.jp/kegg-bin/show_pathway?dme00400
Valine, leucine, and isoleucine biosynthesis	3	Leucine; valine; isoleucine	2.94 × 10^–2^	3.53	1.00	http://www.genome.jp/kegg-bin/show_pathway?dme00290
Nitrogen metabolism	2	Glutamic acid; glycine	5.10 × 10^–2^	2.98	0.00	http://www.genome.jp/kegg-bin/show_pathway?dme00910
Tyrosine metabolism	4	3,4-Dihydroxyphenylglycol; tyrosine; 3,4-dihydroxymandelic acid; noradrenaline	7.44 × 10^–2^	2.60	0.19	http://www.genome.jp/kegg-bin/show_pathway?dme00350
Phenylalanine metabolism	2	Phenylalanine; tyrosine	9.85 × 10^–2^	2.32	0.69	http://www.genome.jp/kegg-bin/show_pathway?dme00360
Alanine, aspartate, and glutamate metabolism	3	Aspartic acid; glutamic acid; 4-aminobutyric acid	1.24 × 10^–1^	2.08	0.55	http://www.genome.jp/kegg-bin/show_pathway?dme00250
Pantothenate and CoA biosynthesis	2	Valine; β-alanine	1.35 × 10^–1^	2.00	0.00	http://www.genome.jp/kegg-bin/show_pathway?dme00770
β-Alanine metabolism	2	β-alanine; aspartic acid	1.54 × 10^–1^	1.87	0.41	http://www.genome.jp/kegg-bin/show_pathway?dme00410
Ubiquinone and other terpenoid-quinone biosynthesis	1	Tyrosine	1.54 × 10^–1^	1.87	0.00	http://www.genome.jp/kegg-bin/show_pathway?dme00130
Pentose and glucuronate interconversions	2	Xylitol; glucose-1-phosphate	1.74 × 10^–1^	1.75	0.00	http://www.genome.jp/kegg-bin/show_pathway?dme00040
D-Glutamine and D-glutamate metabolism	1	Glutamic acid	2.44 × 10^–1^	1.41	1.00	http://www.genome.jp/kegg-bin/show_pathway?dme00471
Purine metabolism	5	Xanthine; hypoxanthine; guanine; 5-aminoimidazole-4-carboxamide; uric acid	2.63 × 10^–1^	1.33	0.07	http://www.genome.jp/kegg-bin/show_pathway?dme00230
Cyanoamino acid metabolism	1	Glycine	2.85 × 10^–1^	1.25	0.00	http://www.genome.jp/kegg-bin/show_pathway?dme00460
Valine, leucine, and isoleucine degradation	3	Valine; isoleucine; leucine	2.95 × 10^–1^	1.22	0.00	http://www.genome.jp/kegg-bin/show_pathway?dme00280
Butanoate metabolism	2	4-Aminobutyric acid; glutamic acid	3.17 × 10^–1^	1.15	0.18	http://www.genome.jp/kegg-bin/show_pathway?dme00650
Riboflavin metabolism	1	*p*-Benzoquinone	3.24 × 10^–1^	1.13	0.17	http://www.genome.jp/kegg-bin/show_pathway?dme00740
Porphyrin and chlorophyll metabolism	2	Glycine; glutamic acid	3.58 × 10^–1^	1.03	0.00	http://www.genome.jp/kegg-bin/show_pathway?dme00860
Methane metabolism	1	Glycine	3.96 × 10^–1^	0.93	0.00	http://www.genome.jp/kegg-bin/show_pathway?dme00680
Glycolysis or gluconeogenesis	2	Lactic acid; glucose-1-phosphate	3.98 × 10^–1^	0.92	0.00	http://www.genome.jp/kegg-bin/show_pathway?dme00010
Glycine, serine, and threonine metabolism	2	Glycine; sarcosine	3.98 × 10^–1^	0.92	0.29	http://www.genome.jp/kegg-bin/show_pathway?dme00260
Galactose metabolism	2	Glucose-1-phosphate; glycerol	4.18 × 10^–1^	0.87	0.05	http://www.genome.jp/kegg-bin/show_pathway?dme00052
Glycerolipid metabolism	1	Glycerol	5.93 × 10^–1^	0.52	0.23	http://www.genome.jp/kegg-bin/show_pathway?dme00561
Starch and sucrose metabolism	1	Glucose-1-phosphate	6.16 × 10^–1^	0.48	0.11	http://www.genome.jp/kegg-bin/show_pathway?dme00500
Propanoate metabolism	1	β-Alanine	6.37 × 10^–1^	0.45	0.00	http://www.genome.jp/kegg-bin/show_pathway?dme00640
Pentose phosphate pathway	1	Ribose	6.57 × 10^–1^	0.42	0.00	http://www.genome.jp/kegg-bin/show_pathway?dme00030
Pyrimidine metabolism	2	2-Deoxyuridine; β-alanine	6.66 × 10^–1^	0.41	0.02	http://www.genome.jp/kegg-bin/show_pathway?dme00240
Pyruvate metabolism	1	Lactic acid	7.24 × 10^–1^	0.30	0.00	http://www.genome.jp/kegg-bin/show_pathway?dme00620
Glycerophospholipid metabolism	1	Ethanolamine	7.83 × 10^–1^	0.24	0.00	http://www.genome.jp/kegg-bin/show_pathway?dme00564
Amino sugar and nucleotide sugar metabolism	1	Glucose-1-phosphate	8.55 × 10^–1^	0.16	0.14	http://www.genome.jp/kegg-bin/show_pathway?dme00520

### Antimicrobial Activity of PBQ

**FIGURE 5:**
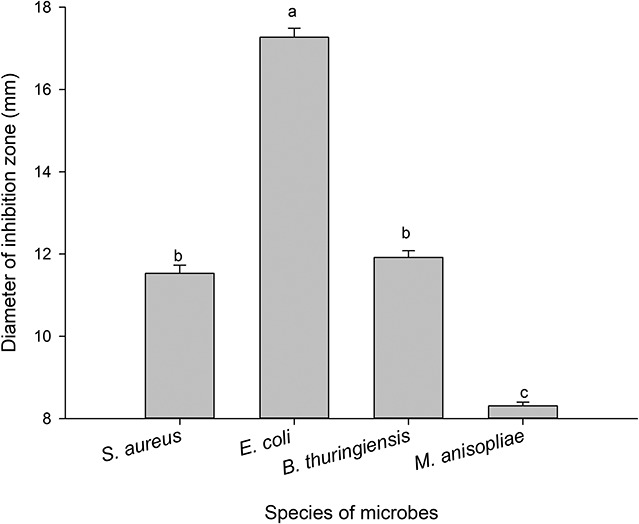
Diameters of inhibition zones against microbes produced by *p*-benzoquinone. The graph showing mean ± standard error was analyzed using one-way ANOVA followed by Tukey’s honestly significant difference (HSD) test. Bars with the same lowercase letters are not statistically different at *P* = 0.05.

The antimicrobial assays of PBQ demonstrated that there were significant differences among different species of microbes (*F*_3_,_356_ = 453.351, *P* < 0.001; Figure 5). The inhibitory activity against *E. coli* and *M. anisopliae* attained the highest level and the lowest level, respectively (Figure 5). The average diameter of the inhibition zones was 17.27 and 8.31 mm for *E. coli* and *M. anisopliae*, respectively (Figure 5). However, the degree of inhibition of growth of *S. aureus* and *B. thuringiensis* did not statistically differ (Figure 5). The MIC against *S. aureus*, *E. coli*, *B. thuringiensis*, and *M. anisopliae* was 0.04, 0.03, 0.04, and 0.16 mg/ml, respectively (Table 5). These results were basically consistent with immunosuppressive efficacy of external secretions produced by RPW, which further confirmed that PBQ was one of the key factors and main active ingredients in external secretions exerting external immune function.

**TABLE 5:** Suppression of microbes exposed to *p*-benzoquinone at various concentrations.

**Concentration (mg/ml)**	**Species of tested microorganisms**			
	***Staphylococcus aureus***	***Escherichia coli***	***Bacillus thuringiensis***	***Metarhizium anisopliae***
10.00	−	−	−	−
5.00	−	−	−	−
2.50	−	−	−	−
1.25	−	−	−	−
0.64	−	−	−	−
0.32	−	−	−	−
0.16	−	−	−	−
0.08	−	−	−	+
0.04	−	−	−	+
0.03	+	−	+	+
0.02	+	+	+	+
0.01	+	+	+	+
0.00	+	+	+	+

*“+”: growth; “−”: inhibition.*

## Discussion

Previous studies have shown that the defensive secretions of a number of coleopteran species from several families, including Tenebrionidae, Silphidae, and Chrysomelidae, exhibited antimicrobial activity *in vitro* (Gross et al., 2008; Cotter et al., 2013; Li et al., 2013; Joop et al., 2014; Pedrini et al., 2015), although, to date, there have been no instances of this being reported in Curculionidae. The present study is the first to demonstrate the immune function of external secretions excreted from RPWs and to further analyze their components and immunologically active constituents. It is also one of a very few reports to study another primary immune system besides the innate internal immunity, i.e., external immunity, which is overlooked in most studies.

In addition to the insect innate barriers posed by the cuticle and internal immune system (Vilcinskas and Götz, 1999; Silva et al., 2016), other factors can limit the effectiveness of pathogens (Pu et al., 2017a). For instance, both ants (Gupta et al., 2015) and bees (Otti et al., 2014) exhibit an array of behavioral and biochemical defenses to prevent pathogenic infection. In some previous studies, insect external secretions were shown to inhibit both bacterial and fungal growth *in vitro*. Secretions from *Tenebrio molitor* can successfully inhibit *E. coli*, *S. aureus*, *Candida albicans*, and *Aspergillus niger* but cannot suppress the growth of *Penicillium citrinum* (Qiang et al., 2006a). The secretion of *Blaps femoralis* has been shown to have antibacterial activity against *E. coli* and *S. aureus* (Li et al., 2009). The antibacterial activity of the anal exudates was also confirmed in burying beetles (Cotter et al., 2013). Salicylaldehyde, released by larvae of the brassy willow leaf beetle, is toxic to *B. thuringiensis* (Gross et al., 2008). (*E*)-2-Hexenal, (*E*)-2-octenal, and (*E*)-2-decenal, three aldehydes from bed bug or stink bug scent glands, are fungistatic toward *M. anisopliae* (Sosa-Gomez et al., 1997; Ulrich et al., 2015). It is clear that these externally secreted compounds are involved in external immunity. Likewise, our results indicated that the external secretions of RPWs inhibited the *in vitro* growth of *E. coli*, *S. aureus*, *B. thuringiensis*, and *M. anisopliae* and may play an important part in disinfecting the RPW’s microenvironment consistent with the action of secretions.

It was reported that the antibacterial activity in larval salivary secretions of *Polistes dominulus* inhibited growth of the gram-positive bacteria *Bacillus subtilis* and the gram-negative bacteria *E. coli* (Turillllazzi et al., 2004). Since then, these insects have often been deemed to be one of the main sources of antimicrobial peptides, having broad-spectrum activity against pathogens, including bacteria, fungi, viruses, nematodes, and some parasites (Pu et al., 2017a; Sewify et al., 2017). Not only has our work established that the external secretions (oral cavity or abdomen) of RPW larvae and adults have antimicrobial activity, we also found a large number of amino acids encoding proteins in the secretions. We speculated that the RPW external immune secretions may contain antimicrobial proteins which can be explored further as a potential source of antimicrobial peptides. However, these will need to be further isolated, purified, and characterized. We were not able to successfully isolate effective antimicrobial peptides from the exocrine secretions in RPW in the present study. There is thus insufficient evidence for the antimicrobial activity of proteins at present. As shown in several studies on other insect species (Qiang et al., 2006a; Joop et al., 2014; Ulrich et al., 2015), the metabolite portion of the external secretions definitely has antimicrobial activity functions based on experimental evidence, e.g., the diameter of antimicrobial inhibition zones produced by some of the potential chemicals including PBQ.

The sensitivity of microorganisms can be attributed to the fact that gram-positive bacteria are surrounded by a cytoplasmic lipid membrane and lack the outer cell membrane which is present only in gram-negative bacteria (Gupta, 2000; Sewify et al., 2017). The absence of this outer membrane usually makes the bacteria vulnerable to the effect of antimicrobial peptides (Gupta, 2011). Interestingly, gram-negative bacteria, namely, *E. coli*, were more sensitive and had considerably larger zones of growth inhibition than gram-positive bacteria, namely, *S. aureus*, after being treated with the RPW secretions in this study. This result is somewhat similar to the findings reported for *T. molitor* (Qiang et al., 2006a), but contrary to those found for *B. femoralis* (Li et al., 2009). It is apparent that multiple factors are present in the secretions, besides antimicrobial peptides, that are capable of interacting with each other to vary their effect on different bacteria.

Li et al. (2009) reported that only the solid phase of *B. femoralis* secretions had bacteriostasis, while the liquid phase did not. Similarly, this phenomenon was also noted in our research. Our results showed that the active compounds that regulated external immunity were mainly concentrated in the solid phase. However, we did find that the solid phase was less responsive to bacteria and fungi than the stoste. In addition, a large assortment of chemicals and their relative amounts were identified and analyzed based on qualitative and quantitative analyses of metabonomics technology. These results, included in this study, further substantiate our supposition that external secretions do not rely on single components to exert their external immunity function but, instead, depend on synergy or antagonism between multiple components to strengthen or weaken their immunosuppressive efficacy. It is quite likely that the antimicrobial activity may be dependent on the interaction of multiple compounds, which may be mixtures of multiple compounds combined in definitive proportions.

Based on metabolome analysis and further antimicrobial functional validation of active components, we concluded that PBQ could be regarded as a key immune defensive secretion of RPWs in resisting the invasion of predators or parasites. This is very similar to the results found in several tenebrionid species. The main defensive secretions produced by *Tribolium castaneum* and *Tribolium confusum* were identified as a mixture of a variety of quinones, including methylquinone, ethylquinone, and benzoquinone, to inhibit the growth of pathogens or repel natural enemies (Alexander and Barton, 1943; Li et al., 2013; Joop et al., 2014; Pedrini et al., 2015; Khan et al., 2016). While Li et al. (2009) reported in another tenebrionid, *B. femoralis*, that 2-methyl-PBQ was one of three predominant secretory compounds. Likewise, it was reported that methyl-PBQ also played a major role in the defense of *T. molitor* (Qiang et al., 2006b).

Quinones, which are one of the major external defensive secretions, are highly reactive, unstable, and toxic and have recently had their biosynthetic pathway preliminarily defined (Pu et al., 2017a). Alkylated benzoquinones are formed by condensation reactions of esters, while PBQ is generated from aromatic rings of amino acids, including tyrosine and phenylalanine (Meinwald et al., 1966; Blum, 1981). In the glandular secretory cells, PBQs are in a form of phenolic β-glucoside contained in the more apical portion, which are then transferred to the inner part of the gland and form active quinones by a series of enzymatic reactions (Happ, 1968). However, the molecular basis for controlling the synthesis and secretion of these secretions remains to be determined, although our research on metabolic pathways in this study will further benefit our understanding of this process.

Insects tend to acquire immune substances directly from other individuals to enhance personal immunity (Otti et al., 2014; Khan et al., 2016), leading to fewer threats in the surrounding environment as a result of bacteriostasis of secretions (Joop et al., 2014; Otti et al., 2014). Therefore, individuals of some species can be attracted to the secretions, although, to some extent, secretions that include quinines, phenols, and aldehydes are toxic to individuals (Pu et al., 2017a). Arthropods have evolved a series of behaviors and mechanisms to reduce the autointoxicative effects of a variety of toxic compounds produced by their species for immune purposes (Blum, 1981). *Tribolium* beetles have the ability to partition the secretions away from the somatic cells, initially, by producing the secretions in cuticle-lined organelles and then retaining them in storage sacs that are formed from invaginations of the cuticle (Roth, 1943; Happ, 1968; Li et al., 2013). In addition, some individuals are known to alert their peers by chemical cues involving secretions to avoid being infected by pathogens (Liang, 1995).

In summary, external secretions including larval oral secretions, larval abdominal secretions, and adult abdominal secretions were collected from *R. ferrugineus*. Functional verification showed that these secretions exhibited potent antimicrobial activity against bacteria and fungi. After analysis of the components of the secretions, PBQ was considered to be the main active substance involved in this process through metabolic profiling. Based on the above analysis, the role that external secretions played in external immune defense was ultimately revealed. Studying the chemical ecology of RPW external immune secretions provides insight into relevant signaling cues, with direct implications for RPW management practices. However, to develop pest inhibitors or behavioral interfering agents for RPWs, the substances acting as the key function and necessary dosage will need to be further explored.

## Data Availability Statement

The datasets for this study can be found in figshare: http://dx.doi.org/10.6084/m9.figshare.9804896.

## Ethics Statement

Ethical review and approval was not required for the animal study because *Rhynchophorus ferrugineus* is exempted from above mentioned requirements.

## Author Contributions

Y-CP and Y-MH designed the research. Y-CP, H-JX, YW, LF, and RW performed the research. Y-CP, H-JX, and X-YL analyzed the data. Y-CP, H-JX, and Y-MH wrote the manuscript. All authors have read and approved the final manuscript.

## Conflict of Interest

The authors declare that the research was conducted in the absence of any commercial or financial relationships that could be construed as a potential conflict of interest.

## Footnotes

1 http://www.omicshare.com

## References

[B1] Al-Ajlan A. M. (2008). “Red palm weevil, *Rhynchophorus ferrugineus* (Olivier) (Coleoptera: Curculionidae),” in *Encyclopedia of Entomology*, Vol. 18 ed. Capinera J. L., (New York, NY: Springer Science Press), 3127–3130.

[B2] Al-Dosary N. M. N. Al-Dobai S. Faleiro J. R. (2016). Review on the management of red palm weevil *Rhynchophorus ferrugineus* Olivier in date palm *Phoenix dactylifera* L. *Emir. J. Food Agric.* 28 34–44.

[B3] Alexander P. Barton D. H. R. (1943). The excretion of ethylquinone by the flour beetle. *Biochem. J.* 37 463–465. 10.1042/bj0370463 16747670 PMC1257939

[B4] Blum M. S. (1981). *Chemical Defenses of Arthropods.* London: Academic Press.

[B5] Brand E. (1917). Coconut red weevil, some facts and fallacies. *Trop. Agric. Mag. Ceylon Agric. Soc.* 49 22–24.

[B6] Chu Y. Zhou F. Zhang M. M. An C. J. (2013). Frontiers of research on the innate immune response in insects. *Chin. J. Appl. Entom.* 50 311–320.

[B7] Cotter S. C. Littlefair J. E. Grantham P. J. Kilner R. M. (2013). A direct physiological trade-off between personal and social immunity. *J. Anim. Ecol.* 82 846–853. 10.1111/1365-2656.12047 23363060

[B8] Cox M. L. (1993). Red palm weevil, *Rhynchophorus ferrugineus*, in Egypt. *FAO Plant Protect. B* 41 30–31.

[B9] Eisner T. (1966). Beetle’s spray discourages predators. *Nat. Hist.* 75 42–47.

[B10] El-Sabea A. M. R. Faleiro J. R. Abo-El-Saad M. M. (2009). The threat of red palm weevil *Rhynchophorus ferrugineus* to date plantations of the Gulf region in the Middle-East: an economic perspective. *Outlooks Pest Manage.* 20 131–134. 10.1564/20jun11

[B11] Faleiro J. R. (2006). A review of the issues and management of the red palm weevil *Rhynchophorus ferrugineus* (Coleoptera: Rhynchophoridae) in coconut and date palm during the last one hundred years. *Int. J. Trop. Insect Sci.* 26 135–154.

[B12] FAOSTAT, (2013). *Food and Agricultural Commodities Production.* Available at: http://www.faostat3.fao.org/download/Q/QC/E (accessed October 23, 2015).

[B13] Fiaboe K. K. M. Peterson A. T. Kairo M. T. K. Roda A. L. (2012). Predicting the potential worldwide distribution of the red palm weevil *Rhynchophorus ferrugineus* (Olivier) (Coleoptera: Curculionidae) using ecological niche modeling. *Fla. Entomol.* 95 659–673. 10.1653/024.095.0317

[B14] Giblin-Davis R. M. Faleiro J. R. Jacas J. A. Peña J. E. Vidyasagar P. S. P. V. Press C. A. B. I. (2013). “Coleoptera: biology and management of the red palm weevil, *Rhynchophorus ferrugineus*,” in *Potential Invasive Pests of Agricultural Crop Species*, ed. Peña J. E., (Wallingford: CABI Press), 1–34.

[B15] Gołebiowski M. Maliñski E. Boguś M. I. Kumirska J. Stepnowski P. (2008). The cuticular fatty acids of *Calliphora vicina*, *Dendrolimus pini* and *Galleria mellonella* larvae and their role in resistance to fungal infection. *Insect Biochem. Mol.* 38 619–627. 10.1016/j.ibmb.2008.03.005 18510973

[B16] Gross J. Schumacher K. Schmidtberg H. Vilcinskas A. (2008). Protected by fumigants: beetle perfumes in antimicrobial defense. *J. Chem. Ecol.* 34 179–188. 10.1007/s10886-007-9416-9 18236110

[B17] Gunawardena N. E. Bandarage U. K. (1995). 4-Methyl-5-nonanol (ferrugineol) as an aggregation pheromone of the coconut pest, *Rhynchophorus ferrugineus* F. (Coleoptera: Curculionidae): synthesis and use in a preliminary field assay. *J. Natl. Sci. Found.* 23 71–79.

[B18] Gupta R. S. (2000). The natural evolutionary relationships among prokaryotes. *Crit. Rev. Microbiol.* 26 111–131. 10.1080/10408410091154219 10890353

[B19] Gupta R. S. (2011). Origin of diderm (Gram-negative) bacteria: antibiotic selection pressure rather than endosymbiosis likely led to the evolution of bacterial cells with two membranes. *Anton. Leeuw.* 100 171–182. 10.1007/s10482-011-9616-8 21717204 PMC3133647

[B20] Gupta S. K. Kupper M. Ratzka C. Feldharr H. Vilcinskas A. Gross R. (2015). Scrutinizing the immune defence inventory of *Camponotus floridanus* applying total transcriptome sequencing. *BMC Genomics* 16:540. 10.1186/s12864-015-1748-1 26198742 PMC4508827

[B21] Happ G. M. (1968). Quinone and hydrocarbon production in the defensive glands of *Eleodes longicolis* and *Tribolium castaneum* (Coleoptera, Tenebrionidae). *J. Insect Physiol.* 14 1821–1837. 10.1016/0022-1910(68)90214-x

[B22] Hou Y. M. Wu Z. J. Wang C. F. (2011). “The status and harm of invasive insects in Fujian, China,” in *Biological Invasions: Problems and Countermeasures*, eds Xie L. H. You M. S. Hou Y. M., (Beijing: Science Press), 111–114.

[B23] Hussain A. Rizwan-ul-Haq M. Al-Ayied H. Ahmed S. Al-Jabr A. M. (2015). Effect of *Beauveria bassiana* infection on the feeding performance and antioxidant defence of red palm weevil, *Rhynchophorus ferrugineus*. *Biocontrol* 60 849–859. 10.1007/s10526-015-9682-3

[B24] Jansson J. Willing B. Lucio M. Fekete A. Dicksved J. Halfvarson J. (2009). Metabolomics reveals metabolic biomarkers of Crohn’s disease. *PLoS One* 4:e6386. 10.1371/journal.pone.0006386 19636438 PMC2713417

[B25] Jaronski S. T. (2010). Ecological factors in the inundative use of entomopathogens. *Biocontrol* 55 159–185. 10.1007/978-90-481-3966-8_12

[B26] Joop G. Roth O. Schmid-Hempel P. Kurtz J. (2014). Experimental evolution of external immune defences in the red flour beetle. *J. Evolution. Biol.* 27 1562–1571. 10.1111/jeb.12406 24835532

[B27] Ju R. T. Li Y. Z. Du Y. Z. Chi X. Z. Yan W. Xu Y. (2006). Alert to spread of an invasive alien species, red palm weevil, *Rhynchophorus ferrugineus*. *Chin. Bull. Entomol.* 43 159–163.

[B28] Khan I. Prakash A. Agashe D. (2016). Immunosenescence and the ability to survive bacterial infection in the red flour beetle. *J. Anim. Ecol.* 85 291–301. 10.1111/1365-2656.12433 26257080

[B29] Lefroy H. M. (1906). *The More Important Insects Injurious to Indian Agriculture.* Calcutta: Government of India Press.

[B30] Li J. Lehmann S. Weißbecker B. Naharros I. O. Schütz S. Joop G. (2013). Odoriferous defensive stink gland transcriptome to identify novel genes necessary for quinone synthesis in the red flour beetle, *Tribolium castaneum*. *PLoS Genet.* 9:e1003596. 10.1371/journal.pgen.1003596 23874211 PMC3708791

[B31] Li W. Ren G. D. Liu F. S. (2009). Chemical composition and antibiotic activity of the defensive secretion of *Blaps femoralis*. *Chin. Bull. Entomol.* 46 424–428.

[B32] Liang Y. S. (1995). The behavior response of *Tribolium castaneum* adults and larvae on the main components of its defensive secretion. *J. Chinese Cereals Oi.* 10:28.

[B33] Liu H. J. Zhao D. Y. Xu J. X. Chen H. M. Huang H. R. (2009). Pest risk analysis of *Rhynchophorus ferrugineus* in Guangdong area. *Guangdong For. Sci. Technol.* 25 20–23.

[B34] Llácer E. Martínez, de Altube M. M. Jacas J. A. (2009). Evaluation of the efficacy of *Steinernema carpocapsae* in a chitosan formulation against the red palm weevil, *Rhynchophorus ferrugineus*, in *Phoenix canariensis*. *Biocontrol* 54 559–565. 10.1002/ps.1882 19924729

[B35] Manachini B. Arizza V. Parrinello D. Parrinello N. (2011). Hemocytes of *Rhynchophorus ferrugineus* (Olivier) (Coleoptera: Curculionidae) and their response to *Saccharomyces cerevisiae* and *Bacillus thuringiensis*. *J. Invertebr. Pathol.* 106 360–365. 10.1016/j.jip.2010.12.006 21147119

[B36] Mastore M. Arizza V. Manachini B. Brivio M. F. (2016). Modulation of immune responses of *Rhynchophorus ferrugineus* (Insecta: Coleoptera) induced by the entomopathogenic nematode *Steinernema carpocapsae* (Nematoda: Rhabditida). *Insect Sci.* 22 748–760. 10.1111/1744-7917.12141 24846780

[B37] Meinwald J. Koch K. F. Rogers J. E. Eisner T. (1966). Biosynthesis of arthropod secretions. III. synthesis of simple p-benzoquinones in a beetle (Eleodes longicollis). *J. Am. Chem. Soc.* 88 341–345.

[B38] Muralidharan C. M. Vaghasia U. R. Sodagar N. N. (1999). Population, food preference and trapping using aggregation pheromone (ferrugineol) on red palm weevil (*Rhynchophorus ferrugineus*). *Indian J. Agr. Sci.* 69 602–604.

[B39] Olson J. F. Moon R. D. Kells S. A. (2009). Off-host aggregation behavior and sensory basis of arrestment by *Cimex lectularius* (Heteroptera: Cimicidae). *J. Insect Physiol.* 55 580–587. 10.1016/j.jinsphys.2009.03.001 19418598

[B40] Otti O. Tragust S. Feldhaar H. (2014). Unifying external and internal immune defences. *Trends Ecol. Evol.* 29 625–634. 10.1016/j.tree.2014.09.002 25278329

[B41] Pedrini N. Ortiz-Urquiza A. Huarte-Bonnet C. Fan Y. Juárez M. P. Keyhani N. O. (2015). Tenebrionid secretions and a fungal benzoquinone oxidoreductase form competing components of an arms race between a host and pathogen. *Proc. Natl. Acad. Sci. U.S.A.* 112 E3651–E3660. 10.1073/pnas.1504552112 26056261 PMC4507192

[B42] Pu Y. C. Hou Y. M. (2016). Isolation and identification of bacterial strains with insecticidal activities from *Rhynchophorus ferrugineus* Oliver (Coleoptera: Curculionidae). *J. Appl. Entomol.* 140 617–626. 10.1111/jen.12293

[B43] Pu Y. C. Hou Y. M. Shi Z. H. Liang X. Y. (2017a). Defensive secretions and the trade-off between internal and external immunity in insects. *Acta Entomol. Sin.* 60 962–974.

[B44] Pu Y. C. Ma T. L. Hou Y. M. Sun M. (2017b). An entomopathogenic bacterium strain, *Bacillus thuringiensis*, as a biological control agent against the red palm weevil, *Rhynchophorus ferrugineus* (Coleoptera: Curculionidae). *Pest Manag. Sci.* 73 1494–1502. 10.1002/ps.4485 27862867

[B45] Qiang C. K. Yang Z. F. Du Y. Z. Tan D. F. Zhang F. S. (2006a). Study on the antimicrobial activity of the defensive secretion of *Tenebrio molitor* L. *Biotechnology* 16 22–24.

[B46] Qiang C. K. Yang Z. F. Zhang S. Y. (2006b). Analysis of chemical constituent in defensive secretions of *Tenebrio molitor* by GC-MS. *Chin. Bull. Entomol.* 43 385–389.

[B47] Roth L. M. (1943). Studies on the gaseous secretion of *Tribolium confusum* Duval. II. The odoriferous glands of *Tribolium confusum*. *Ann. Entomol. Soc. Am.* 36 397–424. 10.1093/aesa/36.3.397

[B48] Sewify G. H. Hamada H. M. Alhadrami H. A. (2017). In vitro evaluation of antimicrobial activity of alimentary canal extracts from the red palm weevil, *Rhynchophorus ferrugineus* Olivier larvae. *Biomed. Res. Int.* 2017:8564601. 10.1155/2017/8564601 28612029 PMC5458366

[B49] Silva F. W. S. Araujo L. S. Azevedo D. O. Serrão J. E. Elliot S. L. (2016). Physical and chemical properties of primary defences in *Tenebrio molitor*. *Physiol. Entomol.* 41 121–126. 10.1111/phen.12135

[B50] Sosa-Gomez D. R. Boucias D. G. Nation J. L. (1997). Attachment of *Metarhizium anisopliae* to the Southern green stink bug *Nezara viridula* cuticle and fungistatic effect of cuticular lipids and aldehydes. *J. Invertebr. Pathol.* 69 31–39. 10.1006/jipa.1996.4619 9028925

[B51] Sun X. Yan W. Qin W. Zhang J. Niu X. Ma G. (2016). Screening of tropical isolates of *Metarhizium anisopliae* for virulence to the red palm weevil *Rhynchophorus ferrugineus* Olivier (Coleoptera: Curculionidae). *Springerplus* 5:1100. 10.1186/s40064-016-2780-6 27468401 PMC4947082

[B52] Tagliavia M. Messina E. Manachini B. Cappello S. Quatrini P. (2014). The gut microbiota of larvae of *Rhynchophorus ferrugineus* Oliver (Coleoptera: Curculionidae). *BMC Microbiol.* 14:136. 10.1186/1471-2180-14-136 24884866 PMC4060583

[B53] Turillllazzi S. Perito B. Pazzagli L. Pantera B. Gorfer S. Tancredi M. (2004). Antibacterial activity of larval saliva of the European paper wasp *Polistes dominulus* (Hymenoptera, Vespidae). *Insect. Soc.* 51 339–341. 10.1007/s00040-004-0751-3

[B54] Turlings T. C. Mccall P. J. Alborn H. T. Tumlinson J. H. (1993). An elicitor in caterpillar oral secretions that induces corn seedlings to emit chemical signals attractive to parasitic wasps. *J. Chem. Ecol.* 19 411–425. 10.1007/BF00994314 24248945

[B55] Ulrich K. R. Feldlaufer M. F. Kramer M. Leger R. J. S. (2015). Inhibition of the entomopathogenic fungus *Metarhizium anisopliae* sensu lato in vitro by the bed bug defensive secretions (*E*)-2-hexenal and (*E*)-2-octenal. *Biocontrol* 60 517–526. 10.1007/s10526-015-9667-2

[B56] Ulrich K. R. Kramer M. Feldlaufer M. F. (2016). Ability of bed bug (Hemiptera: Cimicidae) defensive secretions (*E*)-2-hexenal and (*E*)-2-octenal to attract adults of the common bed bug *Cimex lectularius*. *Physiol. Entomol.* 41 103–110. 10.1111/phen.12129

[B57] Verde G. L. Torta L. Mondello V. Caldarella C. G. Burruano S. Caleca V. (2015). Pathogenicity bioassays of isolates of *Beauveria bassiana* on *Rhynchophorus ferrugineus*. *Pest Manag. Sci.* 71 323–328. 10.1002/ps.3852 24990249

[B58] Vidyasagar P. S. P. V. (1998). *A Brief Report on Red Palm Weevil Research in India.* Available at: http://www.redpalmweevil.com/rpwreport/india.htm (accessed September 30, 2011).

[B59] Vilcinskas A. Götz P. (1999). Parasitic fungi and their interactions with the insect immune system. *Adv. Parasit.* 43 267–313. 10.1016/s0065-308x(08)60244-4

[B60] Wan F. H. Hou Y. M. Jiang M. X. (2015). *Invasion Biology.* Beijing: Science Press.

[B61] Wang G. H. Zhang X. Hou Y. M. Tang B. Z. (2015). Analysis of the population genetic structure of *Rhynchophorus ferrugineus* in Fujian, China, revealed by microsatellite loci and mitochondrial COI sequences. *Entomol. Exp. Appl.* 155 28–38. 10.1002/ece3.3599 29299256 PMC5743574

[B62] Weeks E. N. Logan J. G. Birkett M. A. Pickett J. A. Cameron M. M. (2013). Tracking bed bugs (Cimex lectularius): a study of the effect of physiological and extrinsic factors on the response to bed bug-derived volatiles. *J. Exp. Biol.* 216 460–469. 10.1242/jeb.074930 22996447

[B63] Wei J. N. Shao W. B. Wang X. H. Ge J. Chen X. Y. Yu D. (2017). Composition and emission dynamics of migratory locust volatiles in response to changes in developmental stages and population density. *Insect Sci.* 24 60–72. 10.1111/1744-7917.12396 27554189

[B64] Wei J. N. van Loon J. J. A. Gols R. Menzel T. R. Li N. Kang L. (2014). Reciprocal crosstalk between jasmonate and salicylate defence-signalling pathways modulates plant volatile emission and herbivore host-selection behaviour. *J. Exp. Bot.* 65 3289–3298. 10.1093/jxb/eru181 24759882 PMC4071845

[B65] Wu G. C. Luo X. Y. Heng H. Dong Y. X. Ye L. H. (2007). Risk analysis of alien invasive pest *Rhyncnophorus ferrugineus* (Olivier). *Chin. For. Sci. Technol.* 21 44–46.

[B66] Zada A. Soroker V. Harel M. Nakache J. Dunkelblum E. (2002). Quantitative GC analysis of secondary alcohol pheromones: determination of release rate of red palm weevil *Rhynchophorus ferrugineus*, pheromone from lures. *J. Chem. Ecol.* 28 2299–2306. 12523569 10.1023/a:1021057501459

