# Supplementary material for: External Immune Inhibitory Efficiency of External Secretions and Their Metabolic Profiling in Red Palm Weevil, Rhynchophorus ferrugineus (Coleoptera: Curculionidae)
Source: Front Physiol. 2020 Jan 29;10:1624. doi: 10.3389/fphys.2019.01624 (PMC7025588; doi:10.3389/fphys.2019.01624)
Supplement: Supplementary file 1 [file Data_Sheet_1.pdf]

Supplementary Material

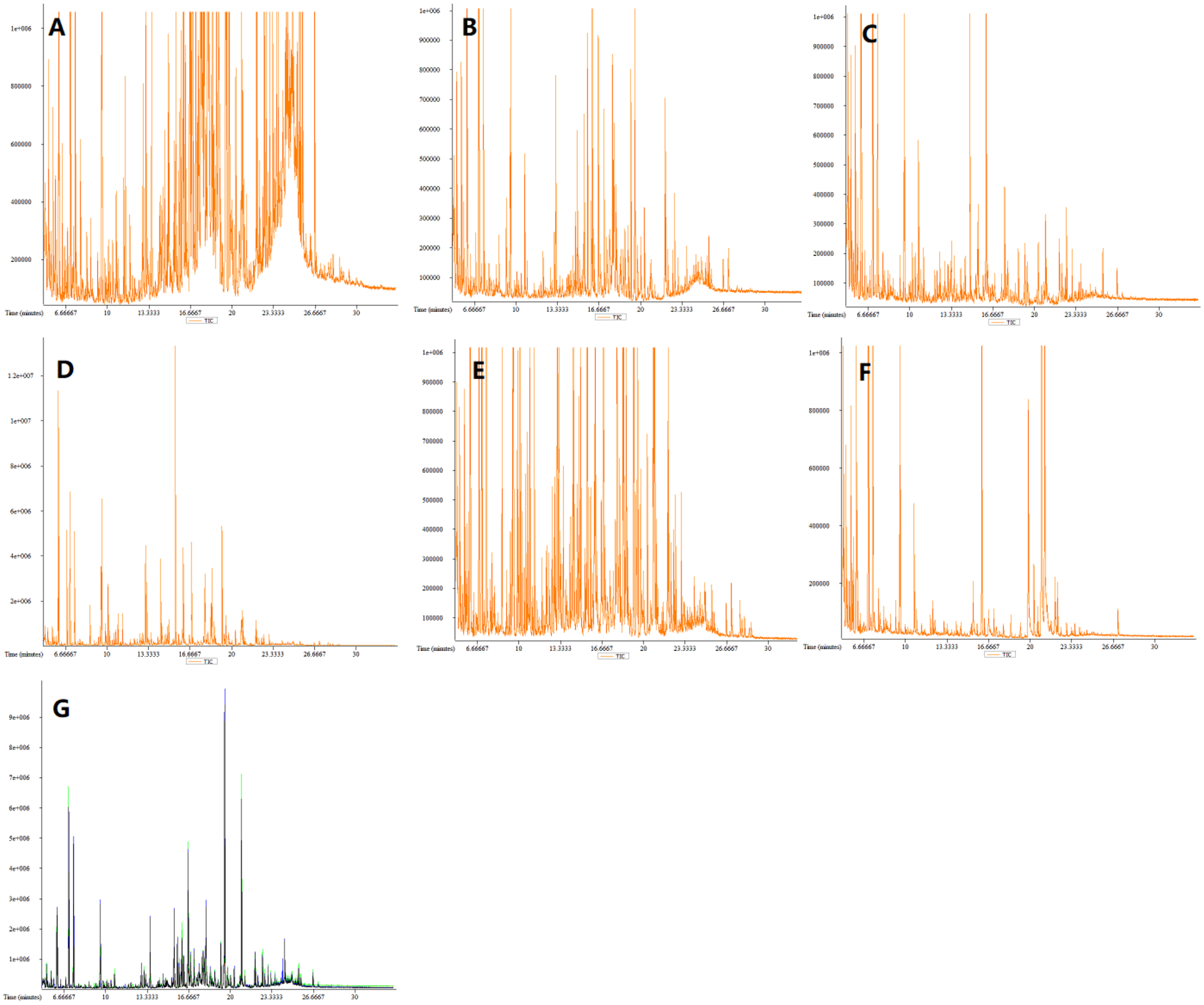

Figure S1 Total ion current (TIC) chromatograms of (A) liquid phase of larval oral secretions, (B) solid phase of larval oral secretions, (C) liquid phase of larval abdominal secretions, (D) solid phase of larval abdominal secretions, (E) liquid phase of adult abdominal secretions, (F) solid phase of adult abdominal secretions, and (G) quality control samples, using gas chromatography–mass spectrometry. All peaks are identified as characterized compounds. Experimental conditions were as follows: sample injection volume, 1 μL; injector temperature, 280 °C; injection mode, pulsed splitless; flow rate, 6.0 mL/min; oven ramp, initial temperature profile began at 60 °C, ramping at 8 °C /min to 125 °C, and then increased to 190 °C at 10 °C /min to 210 °C at 4 °C /min, followed by a programmed rate at 20 °C /min to 310 °C, and 310 °C was finally maintained for 8.5 min.

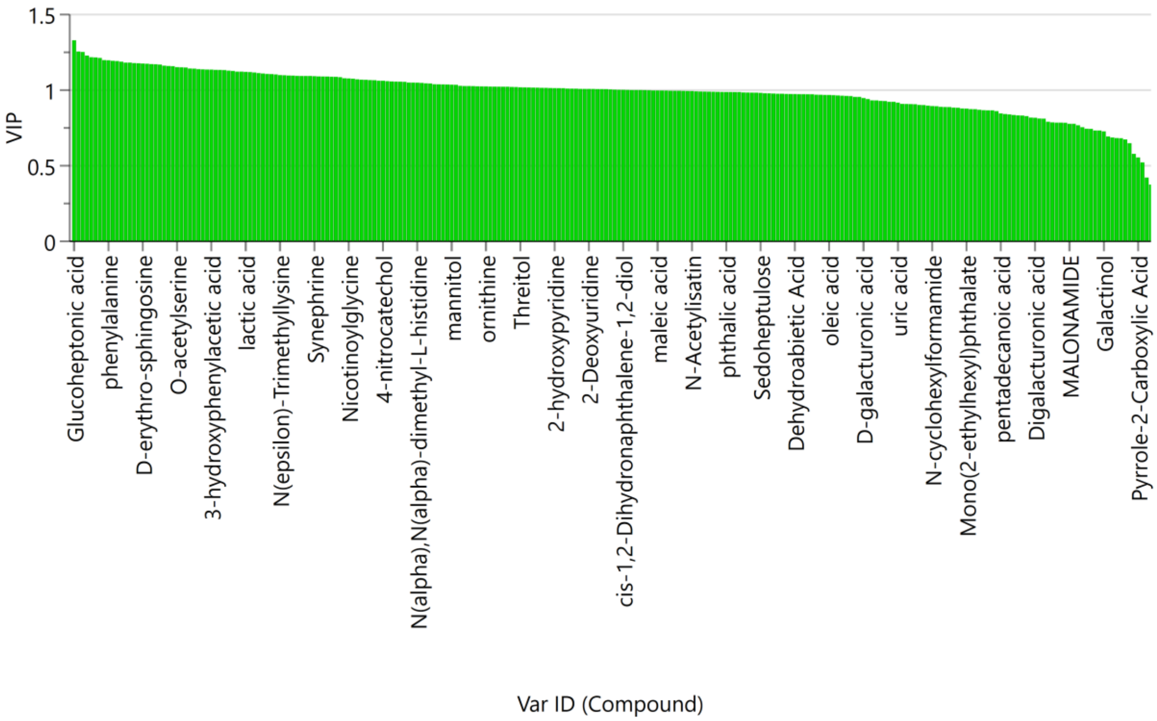

Figure S2 Variables importance of compounds for distinguishing different types of external secretion samples from red palm weevils based on an OPLS-DA model. Compounds with a VIP > 1.0 contributed heavily to the separation of groups. Increases in the VIP value correspond to increases in the contribution of the compound.

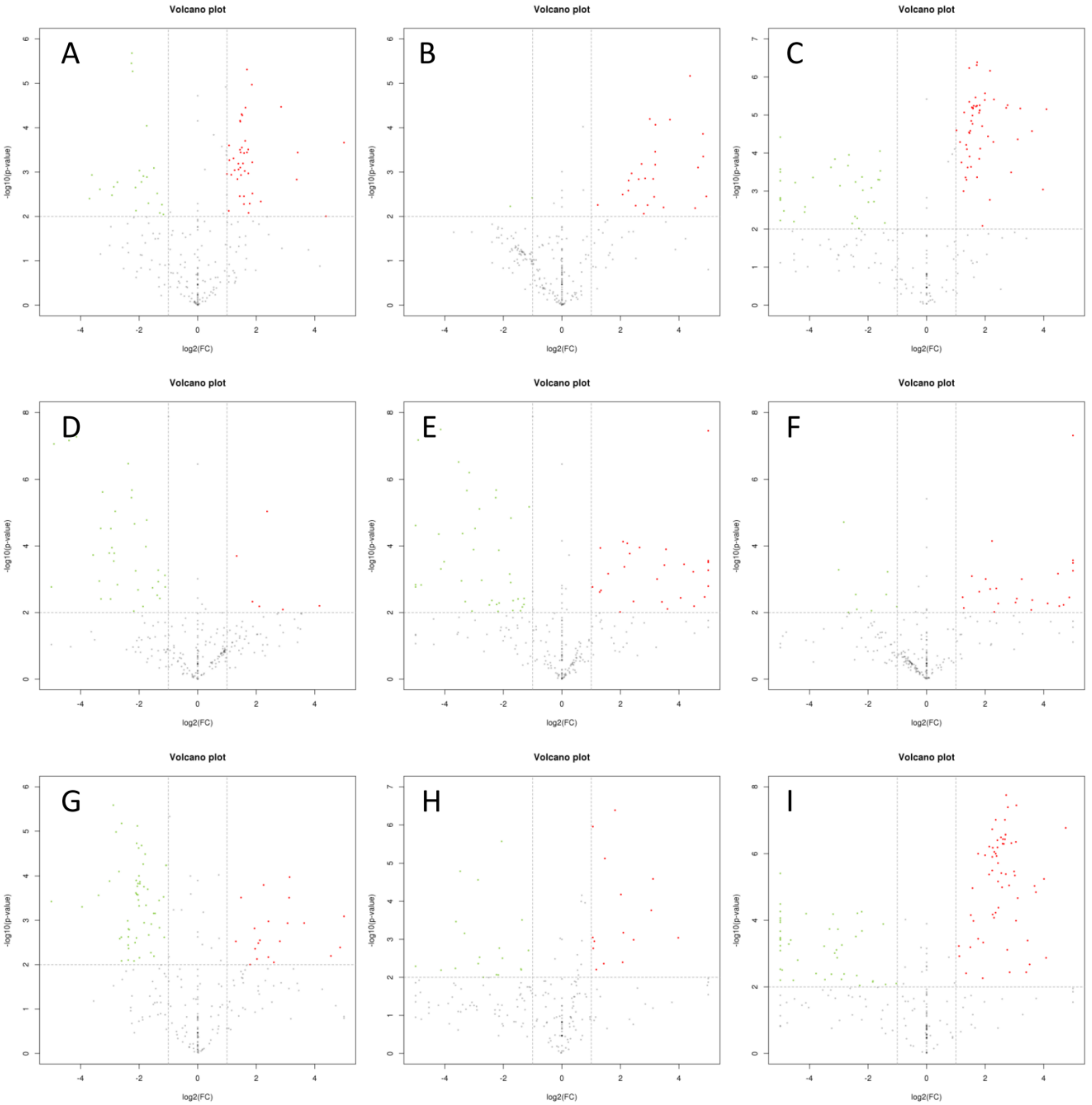

Figure S3 Volcano plots showing all significantly changed differential metabolites between (A) liquid phase and solid phase of larval oral secretions, (B) liquid phase and solid phase of larval abdominal secretions, (C) liquid phase and solid phase of adult abdominal secretions, (D) liquid phase of larval oral secretions and larval abdominal secretions, (E) liquid phase of larval oral secretions and adult abdominal secretions, (F) liquid phase of larval abdominal secretions and adult abdominal secretions, (G) solid phase of larval oral secretions and larval abdominal secretions, (H) solid phase of larval oral secretions and adult abdominal secretions, and (I) solid phase of larval abdominal secretions and adult abdominal secretions. Black dots represent no significant difference between metabolites of the two groups. Red dots represent metabolites with a significant up-regulated content compared to the control. Green dots represent metabolites with a significant down-regulated content compared to the control.

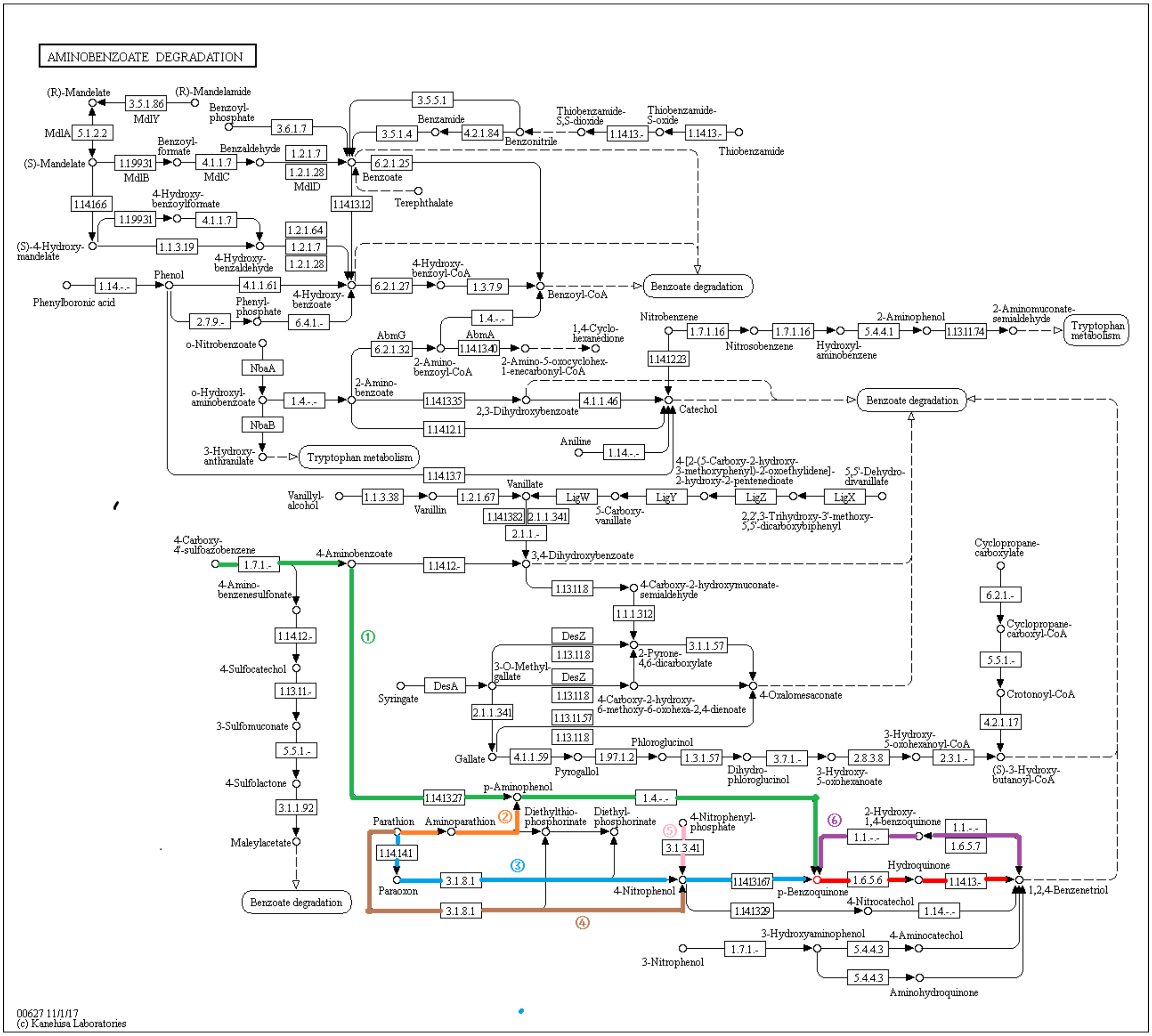

Figure S4 The pathways of aminobenzoate degradation. The red circle represented p-benzoquinone. Lines with color show key metabolic pathways related to p-benzoquinone. The green, orange, blue, brown, pink and purple line indicate six different synthetic pathways for p-benzoquinone, while the red line shows a common pathway involved in catabolism of p-benzoquinone.
